# Traumatic brain injury, neurobiology of aggression and antisocial behavior: from large-scale brain networks to anatomical and molecular substrates

**DOI:** 10.3389/fphar.2026.1925482

**Published:** 2026-10-14

**Authors:** Erica Berretta, Luigi Manni, Marzia Soligo

**Affiliations:** 1 Institute of Translational Pharmacology, National Research Council of Italy (CNR), Rome, Italy; 2 Department of Human Sciences, Università degli Studi Guglielmo Marconi, Rome, Italy

**Keywords:** aggression, amygdala, antisocial behavior, brain networks, lesion network mapping, neuromodulation, traumatic brain injury

## Abstract

Traumatic brain injury (TBI) triggers a dynamic pathological cascade that often culminates in chronic neuropsychiatric sequelae, such as reactive aggression and antisocial behavior. While these debilitating manifestations severely impair social reintegration and quality of life, their heterogeneous nature complicates clinical and forensic characterization. This review addresses this challenge through an integrated, multiscale framework that bridges macro-scale network desynchronization with micro-circuit pathology. We illustrate how the mechanical disruption of major white matter tracts decouples large-scale neurocognitive networks, leaving subcortical emotional anchors in a state of unmodulated hyper-reactivity stripped of prefrontal inhibitory control. The macroscopic untethering operates in synergy with localized cellular failures within specific amygdalar subnuclei, where chronic neuroinflammation, oxidative stress, and ascending neuromodulatory alterations durably shift the affective circuitry toward a hyperexcitable state. By mapping these dimensional neurobiological substrates directly onto the DSM-5-TR taxonomic framework, we spark a new neuroforensic debate regarding criminal responsibility, considering the boundaries of volitional control and the contemporary debate on free will in organically compromised patients. This translational perspective guides the evaluation of novel therapeutic strategies, shifting the paradigm away from non-specific pharmacological sedation toward personalized, state-dependent neuromodulation capable of exogenously restoring circuit synchrony and patient clinical outcomes.

## 1 Introduction

Traumatic brain injury (TBI) is increasingly recognized as a dynamic, evolving pathological condition rather than a static, discrete neurological event (Maas et al., 2008). Beyond the initial mechanical trauma, the long-term sequelae profoundly compromise the patients’ quality of life, acting as a primary barrier to successful social and vocational reintegration (Rauen et al., 2020). The transition from the acute phase is characterized by a progressive remodelling of neural networks, leading to a reduced flexibility in brain state dynamics and increased metabolic cost for network transitions (Roy et al., 2025). Most notably this process involves the structural and functional disruption of the limbic-prefrontal circuit, a network essential for top-down emotional regulation. Central to this framework, amygdala dysregulation emerges as a critical driver of chronic neuropsychiatric deterioration, specifically underpinning the emergence of maladaptive aggression and antisocial behavior (Bickart et al., 2023). Therefore, this review aims to synthesize the current understanding of trauma-induced amygdala dysfunction, examining how altered neurophysiology and connectivity within both local subnuclear microcircuits and large-scale neurocognitive networks contribute to chronic antisocial sequelae. Furthermore, we discuss emerging pharmacological and neuromodulatory strategies targeted at restoring functional connectivity to mitigate post-traumatic aggression, lower recidivism risk, and enhance social reintegration.

## 2 Physiological architecture

The orchestration of complex human behavior, particularly the regulation of affective states, relies on the seamless integration of large-scale brain networks (Yeo et al., 2011; Shine et al., 2019). The Limbic Network (LN), anchored by the amygdala and by the orbitofrontal cortex (OFC), functions as a rapid processor of biological relevance (Palomero-Gallagher and Amunts, 2022; Cocca et al., 2007), promptly detecting environmental stimuli and assigning emotional salience based on innate survival needs (Figure 1). Simultaneously, the Default Mode Network (DMN), anchored by the ventromedial prefrontal cortex (vmPFC) and the posterior cingulate cortex (PCC), provides the necessary social and autobiographical context (Yeshurun et al., 2021). The DMN allows for the ability to infer the intentions of others and evaluate the social appropriateness of one’s actions, integrates this stimulus with social norms and self-referential history (Menon, 2023). These systems operate in tandem with the Salience Network (SN), which comprises the anterior insula (aINS) and the anterior cingulate cortex (ACC). The SN monitors limbic output that deviates from the expected baseline and ensuring that only stimuli requiring behavioral attention trigger the full recruitment of the executive systems (Menon, 2011). The SN signals the need for cognitive appraisal, prompting the Central Executive Network (CEN), encompassing the dorsolateral prefrontal cortex (dlPFC) and the posterior parietal cortex, to intervene (Denny et al., 2023). The CEN is the seat of logical appraisal and cognitive reappraisal (Buhle et al., 2014) and operates by exerting inhibitory control over the LN, dampening reactive impulses (Menon, 2011; Ochsner and Gross, 2005). This hierarchical process allows that emotional arousal remains proportional to the environment and is defined by the efficiency and speed of the CEN-to-LN inhibitory feedback loop. When this loop is intact, the individual possesses the flexibility to transition between internal reflection (DMN) and social engagement (CEN/LN) without impulsive or antisocial derailment (Zilverstand et al., 2017) (Figure 1).

**FIGURE 1:**
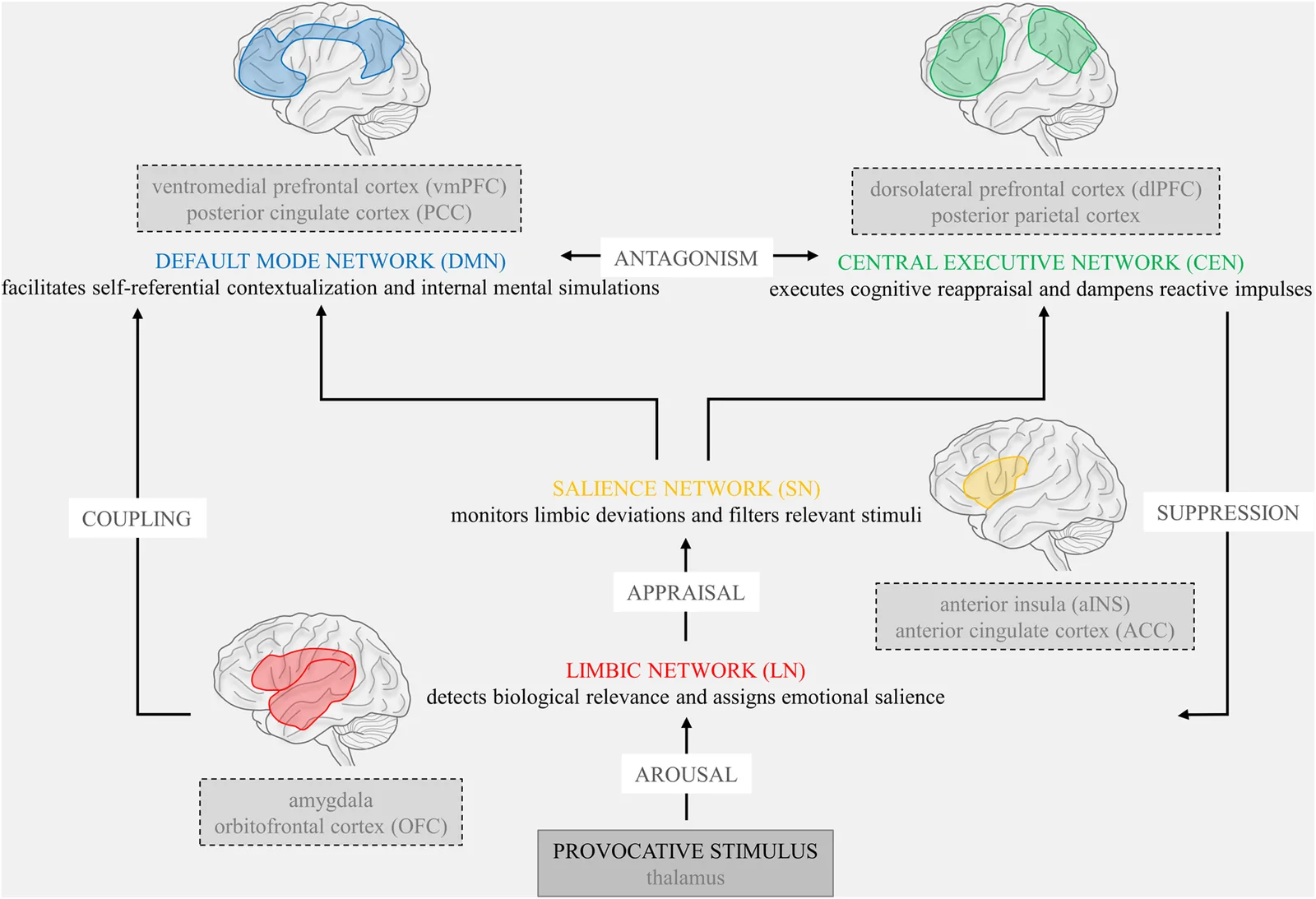
Network integration in affective processing. The diagram illustrates the dynamic, temporal workflow of emotional regulation across major neurocognitive networks. The cascade initiates when a provocative stimulus engages the thalamus, triggering bottom-up arousal within the Limbic Network (LN) (anchored by the amygdala and Orbitofrontal Cortex, OFC) to detect biological relevance. This limbic surge undergoes rapid appraisal by the Salience Network (SN) (comprising the Anterior Insula, aINS, and Anterior Cingulate Cortex, ACC), which functions as a critical homeostatic switchboard. Upon detecting significant homeostatic deviations, the SN orchestrates a dual network shift: it facilitates cognitive engagement by recruiting the Central Executive Network (CEN) (encompassing the dorsolateral Prefrontal Cortex, dlPFC, and Posterior Parietal Cortex) while concurrently driving the suppression of the Default Mode Network (DMN) (anchored by the ventromedial Prefrontal Cortex, vmPFC, and Posterior Cingulate Cortex, PCC) to interrupt internal self-referential simulations. To close the regulatory loop, the activated CEN exerts top-down inhibitory control over the LN, dampening reactive impulses and allowing context-dependent evaluation. Arrows define the directional successional flow, mapping the transition from automatic emotional arousal to cognitive reappraisal.

Having established the macro-scale architecture of affective regulation, it is necessary to descend to the structural and cellular level to understand how this network-level coordination is physically instantiated. The amygdala, as the pivotal node where large-scale network dynamics are translated into discrete behavioral outputs, warrants scrutiny. Its internal nuclear organization and local microcircuit dynamics constitute the anatomical substrate upon which the broader network operates and upon which TBI exerts some of its most consequential effects (McCorkle et al., 2021). The amygdala serves as the structural hub where cortical and subcortical signals converge, undergo integration, and are translated into discrete behavioral outputs. This complex entity comprises at least 13 subnuclei, among which the basolateral (BLA), central (CeA), and medial (MeA) amygdala are the most extensively characterized (Zhang et al., 2021). The BLA, encompassing the lateral (LA) and basal (BA) amygdala, exhibits a cortical-like profile dominated by excitatory glutamatergic neurons and local inhibitory interneurons. In contrast, the CeA and MeA possess a striatal-like architecture, consisting primarily of GABAergic inhibitory neurons (Swanson and Petrovich, 1998). The amygdala’s functional integration relies on a highly organized input-output topography (Figure 2). The LA serves as the sensory gateway, relaying thalamic and cortical information to the BA. Within the BA, these signals converge with context-dependent inputs from the medial prefrontal cortex (mPFC), which provides top-down regulatory control and emotional reappraisal, and ventral hippocampus (vHPC), which furnishes the spatial and temporal context necessary to interpret the affective valence of the stimulus (Phelps and LeDoux, 2005). Thus, the sensory processing within the amygdala is not an isolated event but is instead contextualized through multimodal integration, whereby environmental stimuli are processed in reference to the individual’s autobiographical and social history (Janak and Tye, 2015). Interposed between the BLA and the CeA, the intercalated cell clusters (ITCs) provide an additional layer of GABAergic inhibition, acting as a crucial interface that regulates the threshold for information flow to the CeA (Asede et al., 2022). Finally, the CeA functions as the primary output hub, transmitting processed information to downstream autonomic, somatic, and higher-order cortical sites to coordinate physiological and behavioral responses. Concurrently, the MeA functions as the primary hub for social and reproductive valence (Insel and Young, 2001). Positioned at the intersection of chemosensory and limbic pathways, the MeA integrates direct, pheromonal afferents from the accessory olfactory bulb (Raam and Hong, 2021; Yang et al., 2025), with top-down contextual inputs from the mPFC and vHPC (Figure 2). Serving as a specialized parallel processing and output node, the MeA projects directly to the bed nucleus of the stria terminalis (BNST) and hypothalamic nuclei, thereby gating adaptive social behaviors, defence, and conspecific aggression (Hong et al., 2014). Specifically, its posterodorsal subregion appears to be involved in the expression of aggression, with optogenetic stimulation of GABAergic, but not glutamatergic, neurons promoting aggression and optogenetic silencing suppressing ongoing aggression (Hong et al., 2014).

**FIGURE 2:**
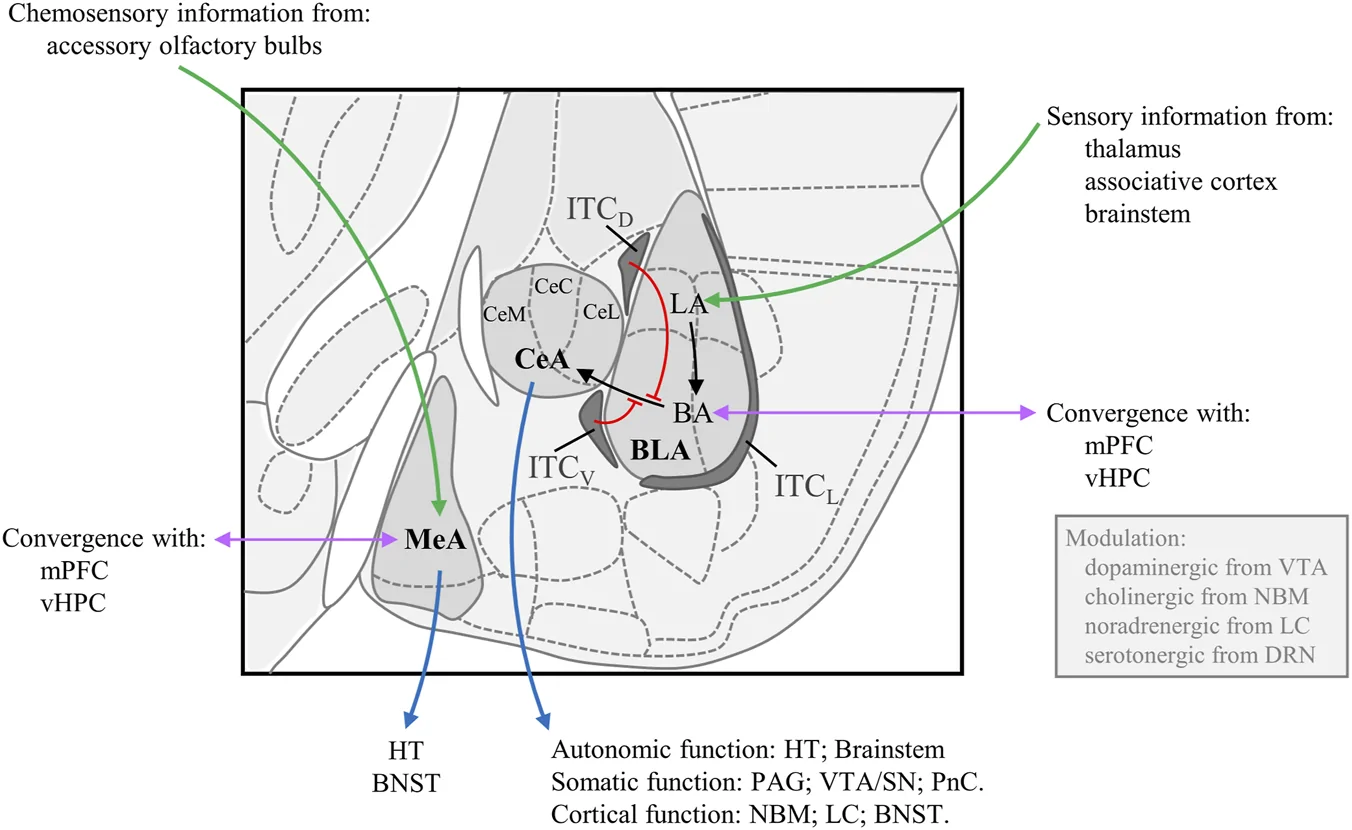
Schematic representation of the amygdala connectivity and nuclear organization. The diagram illustrates the spatial distribution of the primary amygdalar subnuclei and the directional flow of their input-output pathways. Abbreviations: LA, Lateral Amygdala; BA, Basal Amygdala (collectively forming the BLA, Basolateral Amygdala); CeA, Central Amygdala (subdivided into CeL, Central Lateral; CeM, Central Medial; CeC, Central Capsular); MeA, Medial Amygdala; ITC, Intercalated Cell Clusters (D-dorsal; V-ventral; L-lateral). Pathways track the entry of multimodal sensory signals to the LA and chemosensory afferents to the MeA (green arrows), top-down contextual and regulatory projections (pink/purple arrows) from the medial prefrontal cortex (mPFC) and ventral hippocampus (vHPC), local GABAergic feedforward inhibition (red arrows) modulated by the ITCs, and downstream target trajectories (blue arrows). Final outputs are segregated based on functional specialization: the MeA gates social and defensive responses *via* the hypothalamus (HT) and the bed nucleus of the stria terminalis (BNST), while the CeA orchestrates coordinated autonomic (HT, HypoThalamus; brainstem), somatic (PAG, Periaqueductal Gray; VTA/SN, Ventral Tegmental Area/Substantia Nigra; PnC, Reticularis Pontis Caudalis), and higher-order cortical functions (NBM, Nucleus Basalis of Meynert; LC, Locus Coeruleus; DRN, Dorsal Raphe Nucleus).

The functional efficacy of amygdalar processing ultimately depends on the dynamic interplay of synaptic mechanisms operating within its micro-circuit. The BLA operates as a dynamic filter where glutamatergic inputs from sensory and cortical regions are subjected to continuous feed-forward GABAergic inhibition (Szinyei et al., 2000). The fidelity of this signal processing depends on a precise balance between AMPA-mediated fast transmission and NMDA-dependent plasticity (Sah and Lopez De Armentia, 2003). When an affective stimulus is significant, the activation of NMDA receptors triggers Long-Term Potentiation (LTP) in the BLA synapses, reinforcing the emotional memory trace (McKernan and Shinnick-Gallagher, 1997).

The amygdala is dynamically tuned by ascending neuromodulatory projections that recalibrate its firing thresholds. These inputs act as critical gain-control mechanisms: cholinergic inputs from the nucleus basalis of Meynert (NBM) (Tuna et al., 2025) enhance the signal-to-noise ratio by tightening the temporal precision of the neuronal response and promote strength and durability of fear memories (Crimmins et al., 2023), while dopaminergic projections from the ventral tegmental area (VTA) (Sias et al., 2024) scale responsiveness based on incentive salience (Fudge and Emiliano, 2003). Concurrently, serotonergic inputs from the dorsal raphe nucleus (DRN) (Yang et al., 2026) serve as homeostatic modulators that provide an inhibitory bias to facilitate the efficacy of top-down prefrontal control, whereas noradrenergic inputs from the locus coeruleus (LC) (Hao et al., 2025) act as primary arousal signals heightening emotional salience. The amygdala maintains a robust reciprocal connectivity with these structures, as well as with the mPFC and vHPC, to dynamically recalibrate its state for the adaptive regulation of emotional salience (Figure 2).

As illustrated in Figure 3A, cholinergic fibers emanating from the NBM reach the amygdala *via* the stria terminalis and the ventral amygdalofugal pathway acting as a sophisticated gain-control mechanism (Ballinger et al., 2016; Heckers and Mesulam, 1994). Optogenetic evidence highlights that acetylcholine (ACh) release exerts cell-type specific effects that vary with the amygdala’s activity level: in late-firing GABAergic interneurons, cholinergic inputs elicit rapid nicotinic excitatory postsynaptic potentials (EPSPs) that drive local inhibition, whereas they fail to engage fast-spiking interneurons (Unal et al., 2015). The modulatory effect on pyramidal neurons is strictly state-dependent: while ACh induces muscarinic (M1)-mediated inhibitory postsynaptic potentials (IPSPs) in quiescent or low-fire neurons, it switches to an excitatory action in high-fire neurons by prolonging their current-evoked afterdepolarizations (ADPs) (Unal et al., 2015). This dual action serves to increase the signal-to-noise ratio, effectively filtering irrelevant background activity while reinforcing salient emotional signals. Recent findings further elucidate that the cholinergic engagement orchestrates an internal theta rhythm (3–12 Hz), which is essential for synchronizing neuronal ensembles during emotional processing (Bratsch-Prince et al., 2024). The oscillatory state is driven by a specific SOM-CCK microcircuit switch: ACh-mediated activation of M3 muscarinic receptors directly recruits cholecystokinin (CCK)-expressing interneurons, which become the primary drivers of rhythmic inhibition. Crucially, this process is gated by the simultaneous cholinergic inhibition of somatostatin (SOM)-expressing interneurons, which normally suppress CCK cell activity (Bratsch-Prince et al., 2024). By shifting the inhibitory control from SOM-mediated dendritic gating to CCK-driven perisomatic synchronization, ACh creates a permissive window for LTP, ensuring the strength and durability of fear-related memory traces (Jian et al., 2016). This cholinergic sharpening guarantees that the amygdala’s responsiveness is selectively tuned to high-priority threats, preventing emotional over-reactivity to irrelevant stimuli. The cholinergic-induced theta oscillation does not remain confined to the BLA but serves as the temporal substrate for functional coupling with the mPFC and vHPC (Stujenske et al., 2014). By aligning the firing of BLA ensembles with the broader cortical theta rhythm, the amygdala ensures the successful transmission of salient information to the SN, effectively facilitating the transition from local emotional processing to large-scale cognitive evaluation. Although there is currently no direct evidence demonstrating that the MeA undergoes the same acetylcholine-mediated signal-to-noise ratio sharpening, its intrinsic network dynamics are organized around distinct oscillatory frequencies. Specifically, while high-theta oscillations (7–10 Hz) serve as a signature of social arousal that gates conspecific novelty recognition and social memory processing, low-theta rhythmicity (3–7 Hz) is selectively recruited during fear-associated states, orchestrating differential modes of long-range limbic communication depending on the emotional valence of the stimulus (Tendler et al., 2015).

**FIGURE 3:**
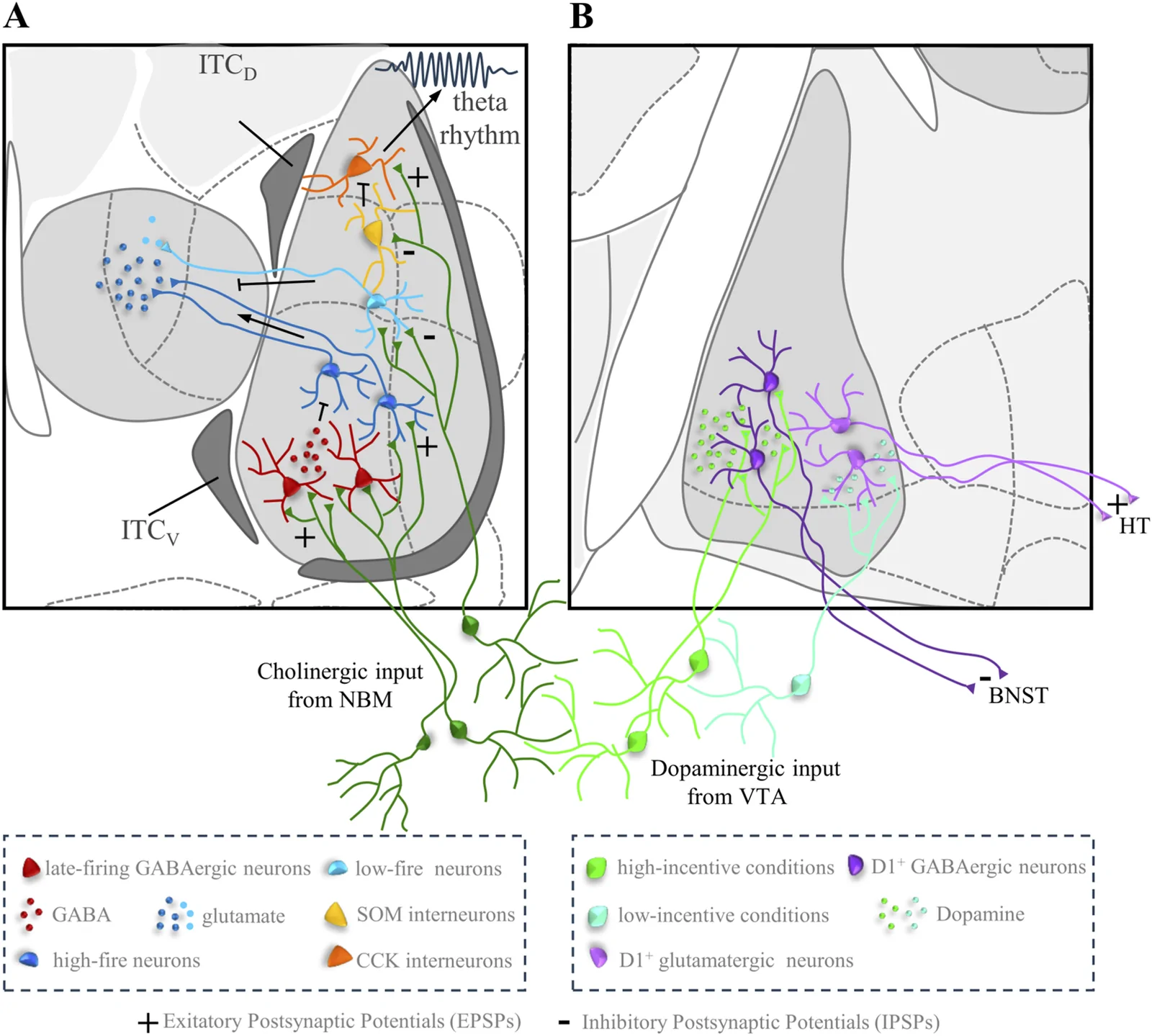
Microcircuit architecture, synaptic plasticity, and neuromodulatory tuning within amygdalar subnuclei. **(A)** The basolateral amygdala (BLA) cholinergic modulation and theta rhythmogenesis. Cholinergic afferents (green neurons) emanating from the nucleus basalis of Meynert (NBM) elicit rapid Excitatory Postsynaptic Potentials (EPSPs; black +) in late-firing GABAergic interneurons (red) to drive robust local GABA release. This cholinergic engagement exerts a cell-type-specific dual action that optimizes the signal-to-noise ratio: it provides a selective Inhibitory Postsynaptic Potentials (IPSPs; black -) that dampens background activity in low-fire neurons (light blue), while enhancing excitability and prolonging afterdepolarizations in high-fire (dark blue) ensembles, by prolonging their current-evoked afterdepolarizations (ADPs). Concurrently, this cholinergic drive orchestrates local network synchrony through a specific somatostatin (SOM, yellow) and cholecystokinin (CCK, orange) interneuronal switch, lifting dendritic gating to create a permissive temporal window for NMDA-dependent Long-Term Potentiation (LTP) and driving the internal theta rhythm (3–12 Hz) necessary for functional coupling with cortical structures. Interposed intercalated cell clusters (ITC) act as the ultimate inhibitory gatekeepers regulating the threshold for information trafficking. **(B)** dopaminergic gating of divergent MeA behavioral outputs. The schematic depicts the synaptic switchboard operation of dopamine D1-receptor-positive neurons within the posteroventral medial amygdala. Under low-incentive conditions (light green neurons), baseline glutamatergic signaling (pink neurons) favours a direct excitatory projection to the hypothalamus (HT; specifically, the ventromedial nucleus), eliciting EPSPs that promote innate defensive avoidance. Conversely, high-incentive social stimuli or conspecific social interactions (fluorescent green neurons) trigger robust ventral tegmental area (VTA) dopaminergic influx; binding to local D1-receptors alters synaptic efficacy, preferentially recruiting a monosynaptic GABAergic projection (violet neurons) to the bed nucleus of the stria terminalis (BNST). This pathway evokes IPSPs in the target nucleus to gate social approach and investigation. Furthermore, this dual-pathway architecture underlies the hardwired organization of aggressive behavior: localized synaptic segregation assigns distinct temporal roles during conspecific conflict, where the MeA-BNST pathway drives attack initiation, while the MeA-Hypothalamus pathway governs the maintenance and persistence of aggressive behavioral output.

Dopamine acts as a versatile modulatory signal that gates synaptic plasticity across the amygdalar complex, simultaneously shaping fear and aggressive behaviors. Specifically, it influences the trafficking of impulses between the BLA and the CeA to calibrate threat responses, while concurrently modulating synaptic efficacy within the MeA to coordinate the switch between social approach and conspecific defensive aggression (de la Mora et al., 2010; Ignacio et al., 2025). Dopaminergic signaling within the amygdala facilitates adaptive threat-coping by neutralizing inhibitory mPFC inputs and modulating local GABAergic microcircuits. By reducing feedforward inhibition, dopamine creates a permissive environment for LTP at excitatory synapses, thereby biasing the recruitment of specific neuronal ensembles that encode fear or safety (Bissière et al., 2003). This modulation appears to be topographically organized: D1 receptors, particularly those highly expressed on Ppp1r1b^+^ reward-responding neurons of the posterior BLA, mediate danger recognition, the retrieval of affective stimulus properties, and the formation of extinction memories (Zhang et al., 2025); D2 receptors in the lateral part of central amygdala (CeL) coordinate brainstem-driven reflex behaviors and adaptive responses to aversive environmental stimuli (de la Mora et al., 2010). Moreover, recent evidence highlights that VTA dopaminergic inputs to the anterior BLA targets Rspo2^+^ fear-responding neurons, where excessive activation can impair extinction and trigger fear relapse (Zhang et al., 2025). As schematized in Figure 3B, preclinical evidence from rodent models demonstrates that the posteroventral subregion of the MeA also receives ascending dopaminergic inputs that regulate either approach or avoidance when encountering innate threat stimuli (Miller et al., 2019). Within the MeA microcircuit, a specialized population of dopamine D1-positive neurons modulates innate defensive and social behaviors through two anatomically and functionally distinct pathways: a predominantly GABAergic, inhibitory projection to the BNST and a glutamatergic, excitatory projection to the hypothalamus (Miller et al., 2019). Circuit-mapping frameworks demonstrate that this divergent architecture regulates the fundamental conflict between exploratory approach and threat avoidance, a process finely tuned by intrinsic dopaminergic signaling where D1 receptor activation dynamically suppresses local IPSPs while gating EPSPs (Miller et al., 2019). Beyond acute threat appraisal, these distinct efferent pathways are differentially weighted to orchestrate the behavioral topography of conspecific aggression. Crucially, these parallel circuits influence separate operational stages of the aggressive response: while the rapid ionotropic IPSPs within the MeA-BNST pathway are primary drivers in gating attack initiation and social approach, the glutamate-mediated field EPSPs recorded within the MeA-Hypothalamus pathway predominantly scales the intensity, persistence, and overall maintenance of the aggressive behavioral output (Miller et al., 2019; Nordman et al., 2020). Under physiological conditions, this precise synaptic segregation allows for the real-time, dynamic gating of innate social responses, ensuring that exploratory interactions and defensive aggression are finely tuned to environmental social cues.

The serotonergic system acts as biochemical facilitator of top-down cortical control (Siever, 2008), wherein dense projections originating from the DRN to the OFC and ACC modulate behavioral outcomes *via* post-synaptic 5-hydroxytryptamine (5-HT, serotonin) receptors (Celada et al., 2013). At the circuit level, the activation of these prefrontal receptor families, predominantly the 5-HT_1A_ and 5-HT_2A_ subtypes, is crucial for driving behavioral inhibition and tuning cortical network dynamics (Celada et al., 2013), effectively suppressing impulsive motor responses while calibrating limbic reactivity to negative emotional stimuli (Siever, 2008). Refining the classical view of a diffuse inhibitory signal, transcriptomic and neuroimaging evidence demonstrates that the heterogeneous brain-wide distribution of serotonin receptor types constitutes a macroscale organizational principle driving behaviorally specific effects across segregated networks (Salvan et al., 2023). Integrating resting-state fMRI with transcriptomic maps, Salvan et al. (Celada et al., 2013) identified a distinct frontolimbic mode of serotonergic variation, anchored in 5-HT_1A_, 5-HT_1F_, and 5-HT_2C_ receptor networks, that specifically governs impulsivity, antisocial problems, and anger-aggression. Providing a structural foundation for this functional segregation, high-resolution *in vivo* positron emission tomography (PET) imaging confirms that the human serotonin system organizes into distinct, spatially homogeneous molecular clusters rather than traditional anatomical borders, establishing that frontolimbic and temporal-mesial structures possess a unique chemoarchitectonic receptor fingerprint (Salvan et al., 2023). The molecular architecture anchoring this frontolimbic network achieves a finely balanced inhibitory tone through opposing but complementary intracellular cascades. The G_i/o_-coupled 5-HT_1A_ and 5-HT_1F_ receptors induce direct neuronal hyperpolarization, dampening pyramidal neuron excitability and threat-related limbic reactivity both post-synaptically and *via* somatodendritic negative feedback (Barnes and Sharp, 1999). Conversely, while the 5-HT_2C_ receptor triggers a fundamentally excitatory G_q_-mediated intracellular cascade, its dense expression on local GABAergic interneurons paradoxically results in systems-level inhibition, suppressing downstream dopaminergic drive within the VTA and mesocorticolimbic pathways (Barnes and Sharp, 1999; Di et al., 2020). Ultimately, the convergent activation of these three receptor subtypes, *via* direct G_i/o_-mediated hyperpolarization and indirect G_q_-mediated GABAergic suppression, constitutes the postsynaptic molecular substrate through which adequate serotonergic tone physiologically maintains behavioral containment and gates reactive aggression.

The LC-norepinephrine (NE) modulatory system represents a central axis in coordinating behavioral and emotional responses to threatening environmental stimuli. From an anatomo-functional perspective, the LC receives substantial corticotropin-releasing factor (CRF) afferents from various brain areas. Among these, the CeA stands out thereby creating a reciprocal activation loop under acute or chronic stress conditions (Buffalari and Grace, 2007; Reyes, 2025). In response to such activation, the LC projects diffusely downstream and one of its primary targets is the BLA (Hao et al., 2025; McCall et al., 2017). Within the BLA, NE acts as a complex neuromodulator, exerting opposing influences *via* different adrenoceptor subtypes. While α2-receptors typically mediate inhibitory tones, the activation of local β-adrenergic receptors increases the excitability of BLA pyramidal projection neurons (Buffalari and Grace, 2007) and facilitates LTP of aversive sensory information, consolidating the traumatic memories and the adaptive fight-or-flight response (Hao et al., 2025). The crucial role of this pathway in acute stress and arousal has been causally proven by *in vivo* optogenetic dissections, which demonstrated that the selective photostimulation of BLA noradrenergic terminals is sufficient to instantly trigger severe anxiety-like behaviors and autonomic flight responses (McCall et al., 2017). Furthermore, when the stressor persists over time, this circuit undergoes pathological dysregulation. Indeed, *in vivo* chemogenetic approaches have demonstrated that when chronically activated, this circuit is susceptible to the transition from sensory pain to the development of generalized anxiety states and exaggerated aversive responses (Llorca-Torralba et al., 2019).

Concurrently, ITCs function as the ultimate inhibitory gatekeepers, controlling the trafficking of impulses. Triggered by top-down glutamatergic input from the mPFC on the NMDA/AMPA receptors within the ITCs micro-circuit (Berretta et al., 2005), these GABAergic clusters release GABA onto CeA neurons, suppressing glutamatergic output and providing the cellular substrate for emotional modulation (Duvarci and Pare, 2014; Likhtik et al., 2008). In the healthy brain, this seamless interplay between cholinergic-driven signal sharpening, NMDA-dependent synaptic plasticity, and prefrontal-driven GABAergic gating ensures that affective responses are not only rapid but also contextually appropriate and modifiable through learning.

## 3 Pathophysiology of post-traumatic dysfunction

The sophisticated and finely tuned neurobiological architecture that governs affective regulation and cognitive control in the healthy brain is exceptionally vulnerable to the mechanical and biochemical forces unleashed by TBI (McAllister, 2011). The transition from physiological harmony to post-traumatic dysfunction begins with the immediate physical disruption of these networks, triggering a cascade of secondary acute and chronic pathophysiological events that fundamentally alter both macro-scale connectivity and micro-circuit homeostasis (Wolf and Koch, 2016).

The structural and functional outcomes of TBI are not merely a consequence of regional neuronal loss but fundamentally stems from a disruption of network synchrony (Wolf and Koch, 2016). Following TBI, the structural breakdown of axonal fibres forces a reorganization of network topology, where the inability to maintain precise temporal coordination between the DMN, CEN, and the limbic structures leads to a failure in effective information integration (Wolf and Koch, 2016). This lack of synchrony effectively decouples the prefrontal regulatory centres from the amygdala, rendering the affective circuitry vulnerable to pathological activity. Specifically, TBI-related Diffuse Axonal Injury (DAI) degrades the structural integrity of white matter, impairing the rapid, real-time communication required for complex cognitive-emotional processing (Sharp et al., 2014). The structural damage is characterized by a significant disruption of functional connectivity between distributed network nodes, resulting in longer average path lengths and reduced global network efficiency (Sharp et al., 2014). A critical component of the failure involves the SN, which is part of the so-called “triple network” proposed by Menon et al. (Menon, 2011), and is implicated in the detection and integration of emotional and sensory stimuli, as well as in modulating the dynamic switch between the internally directed cognition of the DMN and the externally directed cognition of the CEN (Menon, 2011; Bonnelle et al., 2012). Following TBI, the SN often exhibits profound dysfunction, which is directly linked to abnormalities in awareness and self-monitoring (Ham et al., 2014). Specifically, TBI patients frequently display a failure to suppress DMN activity when engaged in externally cued cognitive tasks, a phenomenon associated with significant cognitive performance deficits (Sharp et al., 2014). The inability to pivot between networks essentially traps the brain in an internally focused state, hindering the individual’s ability to interpret external cues, including social and emotional stimuli, through the lens of the CEN. Devoid of functional DMN-mediated context, the individual becomes unable to accurately decode ambiguous social cues, frequently misattributing hostile intent to neutral or benign environmental stimuli (Li et al., 2014). Concurrently, without the regulatory oversight of the OFC, the brain loses its capacity for value-based behavioral selection (Epstein et al., 2016). This network-level desynchronization provides the macro-scale substrate for the disconnection syndrome, where the executive controls provided by the CEN are no longer effectively applied to the limbic structures, leaving the amygdala in a state of unmodulated hyper-reactivity (Ryan et al., 2021). Experimental preclinical evidence confirms this internal network failure, demonstrating that TBI significantly diminishes the propagation of activation from the LA (the primary input) to the CeA (the primary output), effectively weakening internuclear circuit strength (Palmer et al., 2016).

The described functional network failure is underpinned by profound structural damage to the brain’s white matter scaffolds, commonly observed following TBI (Vo et al., 2022; Johnson et al., 2013). Structural integrity of major tracts, including the corpus callosum, cingulum, uncinate fasciculus, and thalamic radiations, is consistently compromised in TBI patients (Dailey et al., 2018). Of particular relevance to affective regulation in humans is the physical injury of the uncinate fasciculus, the primary conduit for the transmission of inhibitory signals from the vmPFC to the amygdala (Von Der Heide et al., 2013). TBI-induced DAI leads to axonal stretching and breakage, creating an anatomical break that renders the prefrontal cortex structurally blind to amygdalar output. In preclinical models, the disruption is observed through the severance of analogous long-range projection fibers, such as the white matter tracts connecting the mPFC and the ventral hippocampus vHPC to the amygdala (Armstrong et al., 2024; Kim and Cho, 2017). Beyond the canonical threat-appraisal pathways centered on the basolateral complex, the disruption of post-TBI large-scale networks also compromises the structural scaffolds dedicated to social survival. Clinical neuroimaging investigations utilizing advanced diffusion tensor imaging (DTI) demonstrate that the stria terminalis, the primary white matter conduit facilitating reciprocal communication between the MeA, the BNST, and hypothalamic nuclei, exhibits significant microstructural degradation, characterized by a marked reduction in fractional anisotropy in chronic TBI patients (Kinnunen et al., 2011). Functionally, this white matter pathology correlates with long-term behavioral alterations in experimental mouse models of TBI, which evaluate post-injury phenotypes through chronic deficits in social interaction paradigms and loss of conspecific social recognition memory (Semple et al., 2012). However, it is worth noting a distinct asymmetry in current neurobiological literature: while the pathophysiological remodeling of BLA microcircuits has been extensively characterized at the single-cell and interneuronal level, the precise intra-MeA cellular post-TBI alterations remain largely under-investigated. Consequently, post-traumatic social impairments are presently understood primarily because of macro-scale white matter disconnection and localized limbic neuroinflammation, rather than resolved microcircuit switches.

As illustrated in Figure 4, following TBI, the balance between glutamatergic excitation and GABAergic inhibition, maintained by a specialized population of parvalbumin (PV)^+^ interneurons, which provide fast, perisomatic inhibition to pyramidal neurons, is selectively vulnerable (Freund and Katona, 2007; Guerriero et al., 2015; Woodruff and Sah, 2007). Converging evidence from preclinical blast models demonstrates a disproportionate loss of PV^+^ interneurons within the BLA, directly implicating GABAergic interneuronal loss as a substrate for post-traumatic hyper-reactivity (Bu et al., 2016). At the molecular level, the selective vulnerability seems to be mechanistically driven by NOX2-derived oxidative stress, which targets PV^+^ interneurons with particular affinity, a phenomenon documented in human post-mortem TBI tissue (Schiavone et al., 2017). The functional consequence is a reduction in GABA-A receptor-mediated inhibitory transmission and a shift in the excitation-inhibition (E/I) ratio that renders BLA principal neurons chronically hyperexcitable (Almeida-Suhett et al., 2014). Compounding this inhibitory deficit, diffuse TBI induces early and persistent dendritic hypertrophy of BLA glutamatergic neurons, expanding the excitatory dendritic surface and further amplifying the network’s capacity for pathological afferent integration (Hoffman et al., 2017).

**FIGURE 4:**
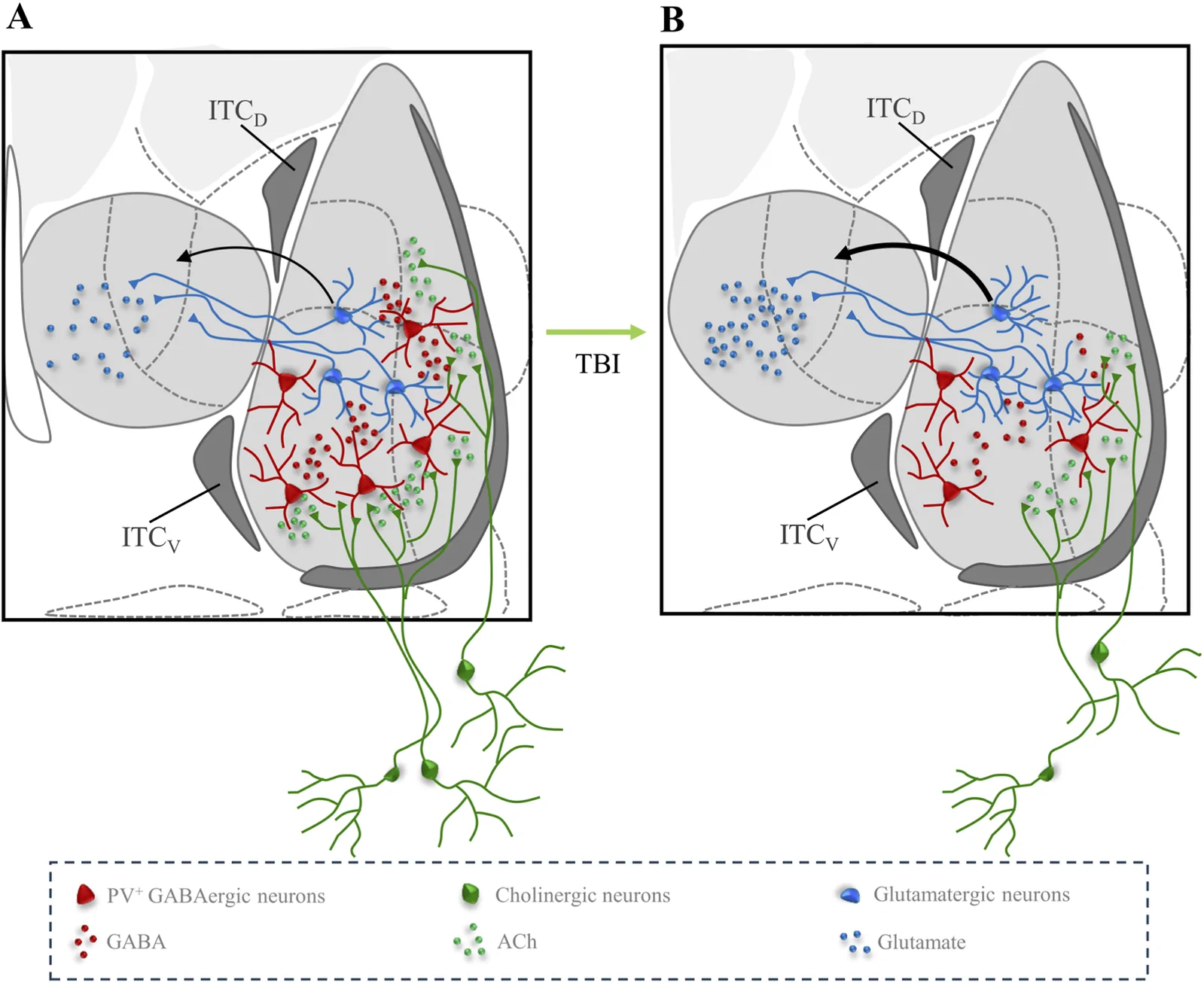
Post-traumatic microcircuit remodeling and synaptic disruption within the amygdala. The diagram illustrates the comparative cellular and neurochemical shift within the basolateral amygdala (BLA) microcircuit from a healthy physiological state **(A)** to post-traumatic brain injury (TBI) dysfunction **(B)**. **(A)** (Baseline/Healthy State): Under physiological conditions, a precise excitation-inhibition (E/I) balance is maintained. Glutamatergic principal neurons (blue) receive continuous, fast perisomatic feedforward inhibition from parvalbumin-positive PV^+^ GABAergic interneurons (red). Concurrently, robust cholinergic drive (green) originating from the nucleus basalis of Meynert (NBM) releases acetylcholine (ACh; green dots) to tune local interneuronal gating, optimizing the signal-to-noise ratio and orchestrating the rhythmic theta oscillations necessary for functional frontolimbic coupling. The dorsal and ventral intercalated cell clusters (ITC_D_ and ITCV) act as intact inhibitory gatekeepers regulating information trafficking. **(B)** (post-TBI State): Following TBI, the structural and neurochemical architecture deteriorates, driving a pathological disinhibition syndrome. Mechanical trauma and oxidative stress lead to a selective, disproportionate loss of PV^+^ GABAergic interneurons (depicted by the depleted red pool), causing a drastic reduction in GABA-mediated local inhibition. Compounding this inhibitory deficit, surviving glutamatergic principal neurons undergo marked dendritic hypertrophy (increase in blue dendrites), expanding their excitatory surface and rendering the BLA chronically hyperexcitable. Vertically, diffuse axonal injury structurally degrades ascending cholinergic projections, resulting in a severe depletion of ACh neurotransmission (loss of green fibers and dots). This cholinergic deprivation abolishes local theta rhythmogenesis, freezing the amygdala in a state of local over-reactivity disconnected from prefrontal oversight. The top-down prefrontal drive deafferentates and silences the inhibitory ITC gate. A concurrent impairment in glutamatergic reuptake mechanism (loss of clearance) leads to a pathological accumulation of extracellular glutamate (blue dots), leaving the CeA constitutively disinhibited and hyper-reactive.

This cellular and structural remodeling is profoundly driven and sustained by a persistent, chronic neuroinflammatory cascade initiated in the acute phase of trauma (Zheng et al., 2022). Following TBI, the mechanical shearing of tissue triggers an immediate and long-lasting activation of resident microglia, shifting them toward a pro-inflammatory phenotype. The activated microglial cells continuously release high concentrations of neurotoxic cytokines, including interleukin-1 beta and tumor necrosis factor-alpha, into the frontolimbic extracellular space (Woodcock and Morganti-Kossmann, 2013; Rathbone et al., 2015). Within the BLA microcircuit, this sustained inflammatory environment acts in synergy with NOX2-derived oxidative stress, creating a hostile metabolic milieu that selectively accelerates the apoptosis of vulnerable PV^+^ interneurons. Beyond direct neurotoxicity, chronic neuroinflammation fundamentally impairs synaptic homeostasis by disrupting astrocytic function (Burda et al., 2016). Under physiological conditions, astrocytes express high levels of excitatory amino acid transporters (primarily EAAT2/GLT-1), which are responsible for the rapid clearance of glutamate from the synaptic cleft (Sheldon and Robinson, 2007). However, post-traumatic microglial cytotoxicity downregulates these transporters, leading to a profound failure in glutamatergic reuptake mechanisms (Guerriero et al., 2015). Within the CeA, the loss of glial clearance causes an abnormal, persistent accumulation of extracellular glutamate, which simultaneously dysregulates local output through a paradoxical reduction in evoked glutamate release followed by a profound failure in neurotransmitter clearance, a distinct signature of chronic synaptic instability recently documented in rodent models of TBI (Beitchman et al., 2020). Beyond the neuroinflammatory and glutamatergic remodeling described above, TBI also compromises the neurovascular unit itself. Preclinical models of mild TBI demonstrate that mechanical injury induces pericyte loss, basement membrane alteration, and blood-brain barrier (BBB) breakdown that precedes astrogliosis and microglial activation, indicating that vascular compromise may act as an early trigger rather than a purely downstream consequence of the neuroinflammatory cascade discussed above (Wu Y. et al., 2021). At the level of cerebral perfusion, longitudinal imaging in rodent models further reveals a delayed, biphasic trajectory: regional cerebral blood flow and vascular morphology partially recover in the subacute phase before undergoing a secondary decline months after injury, a pattern that closely tracks the chronic emergence of social and behavioral deficits (Singh et al., 2024). As a comprehensive treatment of blood-brain barrier and neurovascular unit pathology after TBI falls outside the primary scope of this review, we flag this dimension here as a critical, currently underexplored contributor to post-traumatic network dysfunction that warrants dedicated review elsewhere. Although direct clinical evidence of post-TBI ITCs dysfunction remains limited, it is highly plausible that the structural severance of prefrontal-amygdalar projections effectively silences the ITCs gate by depriving it of the prefrontal glutamatergic drive necessary for its activation (Von Der Heide et al., 2013). This functional deafferentation, coupled with the documented loss of BLA GABAergic interneurons, renders the CeA constitutively disinhibited, thereby providing a cellular substrate for the chronic hyper-reactivity observed in TBI (McCorkle et al., 2021). Building upon this failure of top-down inhibition, the TBI-induced selective severance of projections originating from the vHPC further degrades the system (Atkins, 2011). Under physiological conditions, these inputs serve as a critical spatial and temporal filter, providing the amygdala with the contextual information necessary to calibrate fear responses to the environment. Following TBI, the combination of structural tract degradation and chronic hippocampal atrophy (Harris et al., 2019) effectively blinds the amygdala to contextual cues. The loss of contextual gating forces the amygdala to operate in a default, high-arousal state, where sensory inputs are processed devoid of their situational history. The resulting chronic synaptic instability and network hyper-reactivity chronically bias the affective circuitry toward a maladaptive, hyperexcitable configuration (Coccaro et al., 2023).

This microenvironmental derangement is further compounded by the failure of ascending neuromodulatory systems, particularly the cholinergic projections originating from the NBM (Figure 4). Human post-mortem studies and functional neuroimaging after TBI demonstrate a severe, long-term depletion of cholinergic neurotransmission driven by the chronic loss of ACh neurons within the NBM (Shin and Dixon, 2015). This systemic cholinergic deficit directly derails the temporal architecture of intra-amygdalar communication. The fine acetylcholine-driven microcircuit switch, described in the healthy brain, whereby muscarinic activation tunes local interneuronal populations to orchestrate an internal theta rhythm (3–12 Hz), is disrupted due to the deprivation of primary cholinergic drive. Extracellular electrophysiological recordings in preclinical models of diffuse TBI confirm the network failure, showing a substantial loss of theta-band oscillations and disrupted phase-locking synchronization between the BLA, the mPFC, and the vHPC during emotional processing (Wolf and Koch, 2016). Stripped of this coherent oscillatory substrate for functional coupling, the amygdala can no longer successfully transmit salient information to the SN. This desynchronization effectively traps the system in a state of local emotional over-reactivity, blocking the essential transition to higher-order cognitive evaluation and prefrontal-driven cognitive reappraisal.

The default, high-arousal configuration of the affective circuitry is further exacerbated by the post-traumatic dysregulation of ascending dopaminergic pathways. As documented across both human and preclinical literature, the physiological changes in the dopaminergic system after TBI are closely linked to the direct mechanical damage of projection pathways originating from brainstem nuclei, specifically the VTA and the substantia nigra pars compacta (Mata-Bermudez et al., 2024). TBI structurally disrupts these critical tracts, leading to an interruption of neuronal circuits within the mesolimbic and mesocortical pathways, which in turn causes a severe loss of dopamine homeostasis and establishes a chronic hypodopaminergic state (Mata-Bermudez et al., 2024). At the functional level, because these pathways are responsible for distributing dopaminergic innervation to subcortical and cortical structures, including the amygdala and prefrontal regulatory areas, their disruption directly drives the pathogenesis of post-traumatic neuropsychiatric sequelae (Mata-Bermudez et al., 2024). Crucially, the chronic hypodopaminergic state profoundly derails cost-benefit computations and value-based reinforcement learning. In the TBI brain, dopamine depletion effectively blunts this predictive signaling, leaving the individual hypersensitive to immediate, short-term rewards and disrupting the cognitive evaluation of long-term consequences (Mata-Bermudez et al., 2024).

The translation of mechanical trauma into chronic neurochemical and behavioral sequelae is also deeply rooted in the structural vulnerability of the midbrain. As demonstrated by computational finite element modeling and DTI studies, rotational and acceleration-deceleration forces during TBI impart excessive shear and torsional stress specifically within the DRN (O’Connell et al., 2024). Because serotonergic neurons originating from these midbrain hubs possess exceptionally long, thin, and unmyelinated axonal projections, they are uniquely susceptible to traumatic axonal shearing and subsequent DAI, severely compromising the synthesis and interstitial availability of serotonin (5-hydroxytryptamine, 5-HT). Consequently, the physical dismantling of these ascending fibers induces a profound post-traumatic uncoupling of the corticolimbic network, chronically depriving the prefrontal regulatory structures (such as OFC and ACC) of their primary inhibitory neuromodulator. Deprived of this crucial serotonergic tone, the prefrontal “brake” loses its capacity to modulate subcortical drive, shifting the post-TBI clinical phenotype toward heightened affective hyper-reactivity (O’Connell et al., 2024).

The post-traumatic neurochemical dysregulation extends to the noradrenergic system through a mechanism that mirrors, yet diverges critically from, the serotonergic failure described above. The LC is anatomically positioned in a region subject to peak shearing forces during rotational head acceleration, and its projection neurons are composed predominantly of thin, unmyelinated axons that are selectively vulnerable to traumatic axonal injury (Tsuda et al., 2025; Horvat et al., 2024). Quantitative analysis in preclinical models of TBI documents a robust loss of LC neurons, accompanied by a reduction in noradrenergic fiber innervation (Tsuda et al., 2025). Functionally, this structural damage translates into a significant reduction of basal NE efflux within the mPFC with deficits that accumulate with repeated injuries (Horvat et al., 2024). Critically, TBI triggers a paradoxical, bidirectional neuroplastic reorganization of the central noradrenergic system. While LC neuronal loss reduces noradrenergic tone, the surviving neurons of the LC undergo a disarranged process of regenerative axonal sprouting (Tsuda et al., 2025). The loss of LC-derived noradrenergic input to the CeA removes a modulatory signal that normally gates amygdalar output; the maladaptive sprouting directed toward the BLA, the BNST, and hypothalamic stress-integration nuclei pathologically activates the HPA axis and sustains a state of progressive, chronic hypervigilance and anxiety. Thus, a functional depletion of NE in prefrontal cognitive networks, running parallel to a structural hyper-innervation and β-adrenergic over-activation of limbic fear circuits, provides a compelling neurobiological framework explaining the debilitating comorbidity of cognitive deficits and chronic anxiety in the aftermath of trauma (Giustino et al., 2020).

## 4 The neurobiological foundations of post-traumatic aggression and antisocial behavior

Neuropsychiatric sequelae represent a major burden following TBI, with prevalence rates ranging from 34% in mild to 49% in moderate-to-severe cases within the first year post-injury (Liu and Yi, 2024). Affected individuals experience a widespread constellation of neuropsychiatric and behavioral symptoms, including mood and anxiety disorders (e.g., depression) and emotional dysregulation; apathy, irritability, aggression, impulsivity; altered social conduct, obsessiveness, and psychosis (Li et al., 2023; Kim et al., 2007). Historically, early neurobehavioral nomenclature proposed a fundamental dichotomy segregating these post-traumatic manifestations into “behaviors by excess” and “behaviors by default” (Levin et al., 1992). Under this seminal framework, behaviors by excess (such as impulsivity, agitation, and aggression) were clinically conceptualized as a generalized loss of frontal inhibition, whereas behaviors by default (including apathy, abulia, and emotional flattening) were viewed as a primary failure of behavioral activation (Torregrossa et al., 2023). While this classical taxonomy accurately mapped the overt phenomenological polarization of TBI patients, contemporary clinical neuroscience has transitioned from a purely descriptive paradigm to an integrated, systems-biology model (Stéfan et al., 2016). Modern translational frameworks demonstrate that the active-disinhibited *versus* passive-inhibitory divergence is rigorously dictated by the selective structural topography of large-scale brain network disruption and the concomitant neurochemical and neurotransmitter imbalance.

While acknowledging the clinical significance of both phenotypes, the current review specifically focuses on the active-disinhibited “behaviors by excess” spectrum, owing to its direct and critical implications for forensic psychiatry and the genesis of violent conduct. Reflecting this profound clinical relevance, epidemiological and forensic data indicate that an overwhelming majority of violent offenders (with rates reaching up to 60.2%), have experienced at least one TBI in their lifetime (Colantonio et al., 2007; Maresca et al., 2023; Katzin et al., 2020). Much of the evidence linking TBI to aggression has been indeed derived from forensic, correctional, or clinical populations, which may overrepresent individuals with pre-existing behavioral and psychiatric vulnerabilities. Relatively few studies have investigated the association between TBI and aggression in community-based samples, limiting the generalizability of findings to the broader population (Buckley et al., 2017). Moreover, the causal inference linking TBI to aggression is complicated by several confounding variables that are rarely controlled for across studies. Pre-existing psychopathology, including childhood conduct problems and attention-deficit/hyperactivity disorder, has been identified as a risk factor for both sustaining a TBI and for subsequent aggressive behavior (Schachar et al., 2015), raising the possibility of reverse causation or shared underlying vulnerability rather than a purely mechanical, injury-driven pathway. Substance use, and alcohol in particular, is both a common antecedent circumstance of TBI and a factor that TBI itself may independently promote, representing a further confound seldom disentangled in the existing literature (Weil and Karelina, 2017). More broadly, methodological reviews of the aggression literature note that head-injury studies frequently lack prospective designs and adequate control for developmental risk factors such as prior history of violence and socioeconomic status (Bannon et al., 2015). Consequently, while this review emphasizes the neurobiological substrates of post-traumatic aggression, the described mechanisms should be interpreted as contributing rather than sufficient causal factors, and prospective, confounder-adjusted studies in community-based samples remain necessary to establish the independent contribution of TBI-related neurobiology to aggressive outcomes. Nevertheless, longitudinal evidence suggests that aggressive behavior remains a common and persistent consequence of TBI. In a cohort of 228 individuals with moderate-to-severe TBI assessed at 6, 24, and 60 months following discharge from a specialized brain injury rehabilitation service, approximately one-quarter of participants met criteria for clinically significant aggression at each follow-up time point, indicating a stable burden of post-traumatic aggressive behavior over time (Baguley et al., 2006).

The transition from systemic physiological instability to chronic behavioral dysfunction represents one of the most debilitating trajectories following TBI (Li et al., 2023). While the immediate post-injury phase is characterized by acute biological alterations, the long-term clinical landscape is frequently dominated by a significant incidence of aggressive, violent, and antisocial behaviors (Maresca et al., 2023; Rao et al., 2009; Vaughn et al., 2019) (see Box 1; Box 2). This chronic trajectory is not confined to aggression alone: experimental models similarly demonstrate that TBI-induced social behavioral deficits persist over the long term and can parallel a delayed decline in cerebrovascular perfusion months after injury, further corroborating chronic neurobehavioral dysfunction as a stable, clinically relevant post-traumatic outcome (Semple et al., 2012; Singh et al., 2024). Epidemiological data underscore this shift, revealing that psychiatric sequelae such as verbal and physical aggression are not merely incidental symptoms but are deeply rooted in the permanent structural and functional reconfiguration of the brain (Tateno et al., 2003). Demographic variables seem to have an impact on these results, as individuals who have suffered injuries at a young age show a greater propensity to develop chronic violent behaviors (Baguley et al., 2006). Conversely, although a significant difference in the incidence of head injuries and crash experiences between the sexes is evident (Kort-Butler et al., 2024), data regarding sex-related aggressive behaviors are conflicting, due to historical methodological biases, confounding baseline injury incidence rates, and the complex interplay of pre-morbid functioning and psychiatric comorbidities that frequently obscure direct gender stratification (Kim et al., 2007). Crucially, recent empirical data in emerging adults confirm that when controlling for baseline exposure, a history of head injury increases aggressive conduct symmetrically across both sexes, demonstrating a fundamental phenotypic convergence in long-term behavioral sequelae (Kort-Butler et al., 2024). In this context, translational evidence untangles these discrepancies by demonstrating a critical, sex-dependent interaction between the age at injury and subsequent violent outcomes (Shonka and Hylin, 2025). While pre-adolescent trauma triggers a sharp spike in chronic adult aggression selectively in males, early infant injuries produce a paradoxical, severe behavioral impairment and heightened aggressive drive exclusively in females, driven by a sex-specific disruption of prefrontal-amygdala synaptic remodeling, as shown in a rat model of TBI (Shonka and Hylin, 2025). These findings are even more surprising when compared with the developmental models of idiopathic psychopathy and early-onset stable antisocial disorders, where a significant gender gap and divergent neurobiological pathways prevail (Hodgins, 2020). In those non-traumatic, developmental cohorts, males disproportionately exhibit cold, proactive externalizing violence driven by structural amygdalar hypo-reactivity and callous-unemotional traits; conversely, female antisocial phenotypes are rarer, showcasing a lower propensity for overt physical violence and presenting instead a clinical picture heavily dominated by frontolimbic hyper-reactivity, severe affective dysregulation, and heightened emotional anxiety (Hodgins, 2020). While this non-traumatic gender divergence underscores a distinct constitutional trajectory for idiopathic “behaviors by excess”, it raises critical questions regarding how mechanical trauma dynamically alters these sex-specific developmental thresholds.

Box 1 **Transdiagnostic constructs and dimensional symptoms in antisocial and aggressive phenotypes.**

**Methodological note for the reader**

Unlike categorical psychiatric diagnoses, transdiagnostic constructs represent dimensional, continuous traits and behavioral mechanisms that cut across multiple clinical disorders. In the context of TBI and antisocial conduct, these dimensions serve as the elemental building blocks or clinical proxies that link specific neural network disruptions to overt, heterogeneous behavioral outputs.

**Affective and emotional dimensions**

**Callousness (lack of empathy)**

A profound affective deficit marked by a lack of emotional sensitivity, guilt, remorse, and disregard for the feelings and suffering of others. This dimension is divisible into cognitive empathy (the capacity to understand another’s perspective) and affective empathy (the capacity to vicariously experience another’s emotional state). While developmental psychopathy is characterized by a severe, selective deficit in affective empathy, post-traumatic states can compromise both dimensions through structural disruption of the ventromedial moral and mentalizing networks (Hernandez-Lallement et al., 2018).

**Emotional dysregulation**

The structural inability to modulate the intensity, duration, or expression of explosive emotional states, particularly anger and anxiety. Following TBI, it typically arises from the anatomical deafferentation between orbitofrontal/ventromedial prefrontal nodes and the limbic system, rendering the individual incapable of deploying cognitive reappraisal strategies to mitigate emotional surges.

**Irritability**

A chronic affective state characterized by a pathologically lowered threshold for anger and frustration in response to minimal environmental stimuli. Unlike acute rage, irritability is a persistent, underlying emotional reactivity. It is increasingly conceptualized as a transdiagnostic dimension associated with emotional dysregulation, reactive aggression, and altered threat-processing circuitry (Leibenluft, 2017). Neurobiologically, it reflects a state of baseline tonal hyper-arousal and poor metabolic buffering within the frontolimbic circuits, serving as the immediate emotional fuel for reactive aggression.

**Sensation seeking**

The active pursuit of varied, novel, complex, and intense sensations and experiences. Sensation seeking describes the inclination of certain individuals to deliberately seek out situations and experiences that offer high levels of stimulation and novelty, even in the face of potential dangers or negative consequences (Roberti, 2004; Zuckerman and Wright, 2015).

**Cognitive and inhibitory dimensions**

**Impulsivity**

A multi-faceted behavioral trait defined by a marked tendency to act rapidly and without premeditation, forethought, or consideration of long-term negative consequences (Evenden, 1999). Clinically, it encompasses both motor impulsivity (the failure to inhibit a prepotent behavioral response) and cognitive impulsivity (an inability to delay gratification or compute reward-to-risk ratios). It is the clinical hallmark of executive dysfunction and frontal lobe disinhibition (Dalley et al., 2011; Bari and Robbins, 2013).

**Hostile attribution bias.**

A social-cognitive distortion wherein an individual consistently interprets ambiguous or benign behaviors of others as intentionally hostile, provocative, or threatening. This bias acts as a pathological cognitive filter, misrouting neutral environmental cues directly into the salience network, thereby triggering defensive, reactive aggressive outbursts (Yeager et al., 2013).

**Behavioral output dimensions**

**Violence**

The intentional use of physical force or power against oneself, another person, or a group/community that results in, or has a high likelihood of resulting in injury, death, psychological harm, or deprivation (Organisation mondiale de la santé, 2002). The extreme physical, destructive, and coercive endpoint of the aggressive spectrum, violence represents a severe behavioral expression of aggressiveness, although aggressiveness does not necessarily culminate in violent behavior. While aggression constitutes a broad neurobiological construct encompassing verbal hostility, social dominance, and competitive drive, violence is strictly operationalized in forensic and clinical settings as the explicit execution of severe, physically damaging force directed against individuals, objects, or societal structures.

**Antisocial behavior**

A broad, non-diagnostic behavioral umbrella that encompasses any act violating social norms, legal codes, or the fundamental rights of others. This dimension exists on a continuum of severity, ranging from low-level social nuisances and deceitfulness to severe criminal intimidation, property destruction, and structural vandalism.

Box 2 **Clinical, psychiatric, and neuro-forensic taxonomy of aggressive and antisocial behaviors.**

**Methodological note for the reader**

To navigate the complex literature on antisocial conduct, it is clinically essential to distinguish among three distinct taxonomies:

**General Clinical Phenotypes**

Concepts defined within clinical neuroscience to classify types of aggressive actions based on behavior and physiology.

- Diagnostic and Statistical Manual of Mental Disorders (DSM-5-TR) (DSM Library)
- Disorders: Official, standardized psychiatric diagnoses, which are sub-divided into general clinical syndromes (e.g., IED) and rigid, pervasive Personality Disorders (e.g., Antisocial personality disorder - ASPD as the framework for acquired antisocial shifts).

**Forensic and Theoretical Constructs**

Clinical profiles and syndromes born within psychology and behavioral neurology that are not standalone diagnoses in the DSM-5-TR but are widely validated in literature and forensic settings.

**Clinical phenotypes of aggression**

**General neuropsychiatric definition**

In clinical neuroscience, aggression is defined as a heterogeneous suite of overt behaviors characterized by the intent to cause physical or psychological harm to individuals, objects, or social structures, irrespective of whether the behavior is impulsive or premeditated (Anderson and Bushman, 2002). It reflects a profound breakdown in emotional self-regulation and behavioral inhibition. The DSM-5-TR does not classify aggression as a standalone disorder or a distinct diagnostic entity; instead, the manual operationalizes it as a transdiagnostic behavioral criterion (a symptom) that must be evaluated based on its frequency, intensity, and underlying motivational drivers across different psychiatric conditions.

**Behavioral phenotypes: the functional dichotomy**

**Reactive (or Impulsive/Affective) aggression**

An emotional, hostile, and defensive response to a perceived threat, frustrating environmental stimulus, provocation, or social rejection. It is typically driven by anger, high autonomic arousal (sympathetic surge), poor inhibitory control, and a catastrophic failure of top-down cortical braking systems (OFC/vmPFC) to modulate a hyper-reactive amygdalar core (BLA/CeA). This is the primary phenotype triggered by diffuse axonal injury (DAI) in TBI (Raine et al., 2006; Blair, 2016).

**Proactive (Instrumental/Predatory) Aggression**

A cold, deliberate, calculated, unprovoked, and goal-directed behavior executed to obtain a specific reward, dominance, coercion, status, or predatory advantage. It occurs under low autonomic arousal and reduced emotional responsivity. It is directed by baseline amygdala hypo-reactivity, paired with an intact but emotionally detached Central Executive Network (CEN) that plans the action without empathetic constraints. Unlike reactive aggression, proactive aggression is generally associated with callous-unemotional and psychopathic traits (Cupaioli et al., 2021; Romero-Martínez et al., 2022) (see below in the box) and could emerge post-trauma only through highly localized lesions of the moral prefrontal nodes (vmPFC).

**DSM-5-TR diagnostic categories (official psychiatric taxonomy)**

Since aggression is treated as a symptom rather than a disease, the DSM-5-TR anchors it within specific diagnostic frameworks depending on its etiology:

**Impulse-control and conduct framework (non-personality disorders)**

**Intermittent Explosive Disorder (IED)-[Primary/Impulsive Framework]**

A clinical diagnosis where recurrent, non-premeditated aggressive outbursts constitute the core feature of the disorder. The explosive rage is strictly reactive and represents a primary failure to control aggressive impulses under psychosocial provocation.

**Conduct Disorder (CD) and Callous-Unemotional (CU) Traits-[Developmental Framework]**

Conduct Disorder represents the childhood and adolescent precursor to adult ASPD, defined by repetitive behaviors that violate basic social rules and the rights of peers (American Psychiatric Association, 2022). When specified with Callous-Unemotional (CU) traits, it denotes a severe developmental subtype marked by a structural lack of empathy, guilt, and emotional sensitivity. DSM-5-TR includes the specifier “with limited prosocial emotions,” reflecting the presence of callous-unemotional traits (Frick and White, 2008). This specific phenotype is strongly tied to proactive aggression and serves as the primary developmental proxy for adult psychopathy.

**Personality framework (official DSM-5-TR personality disorders)**

**Antisocial personality disorder (ASPD)-[Adult framework]**

In the DSM-5-TR, ASPD is defined as a pervasive psychiatric pattern of disregard for, and violation of, the rights of others, with evidence of conduct disorder, beginning in childhood or early adolescence and continuing into adulthood. Core clinical features include chronic impulsivity, deceitfulness, systematic irresponsibility, persistent irritability, aggressiveness, irresponsibility, and a profound lack of remorse. While primary ASPD is a developmental personality construct that constitutes a psychiatric diagnosis, antisocial behavior represents a broad behavioral construct encompassing acts ranging from nuisance behavior to intimidation and vandalism.

**Personality Change Due to Another Medical Condition (PCDAMC, Aggressive Type)-[Secondary/Acquired Framework]**

The definitive diagnostic category for chronic TBI sequelae. It specifies a persistent and structural alteration in an individual's prior personality pattern, where the predominant clinical feature is a marked inability to control aggressive impulses. Because this shift is directly attributable to the physiological consequences of brain trauma, it provides the official psychiatric framework for what is historically termed “acquired sociopathy”.

**Forensic and theoretical constructs (non-DSM taxonomy)**

**Psychopathy**

A severe personality construct evaluated via the Psychopathy Checklist-Revised (PCL-R) developed by Dr. Robert D. Hare (Brazil and Forth, 2016). Unlike ASPD (which focuses heavily on behavioral and criminal acts), psychopathy is defined by a specific cluster of interpersonal and affective traits, including superficial charm, grandiosity, pathological lying, manipulation, callousness, and a structural incapacity for empathy or guilt (Hare and Neumann, 2008). Contemporary models distinguish affective-interpersonal features from impulsive-antisocial/lifestyle dimensions and implicate abnormalities in emotional learning, reinforcement processing, and amygdala–vmPFC networks (Patrick et al., 2009; Blair, 2003). Neurobiologically, it is tied to structural hypo-reactivity of the amygdala and blunted autonomic responses, heavily biasing the individual toward proactive aggression.

**Sociopathy**

An informal, non-diagnostic term denoting chronic antisocial patterns that are secondary to environmental factors (e.g., severe childhood neglect, poor socialization) rather than constitutional deficits, often biased toward reactive aggression.

**Acquired sociopathy**

A neuropsychiatric syndrome originally coined by Antonio Damasio to describe a profound, post-morbid identifiable alteration in social conduct, characterized by antisocial, disinhibited, or socially inappropriate behavior and resulting from structural damage to the vmPFC/OFC. It represents the neuro-structural and anatomical equivalent of the official DSM-5-TR diagnosis of PCDAMC. Unlike developmental antisocial personality disorder or psychopathy, acquired sociopathy is conceptualized as secondary to TBI or neurodegenerative disease and may involve impaired moral reasoning, empathy, decision-making, and impulse control (Damasio et al., 1990; Anderson et al., 1999).

The neurobiological mechanisms underlying the post-traumatic structural reconfiguration can be rigorously deconstructed through the dual-network architecture of lesion-behavior mapping. Recent landmark evidence by Miller et al. (Miller et al., 2026) applied Lesion Network Mapping (LNM) to focal brain injuries associated with new-onset aggression, tracing anatomically heterogeneous lesions onto a standardized atlas to evaluate their functional connectivity profiles against resting-state data from healthy human cohorts. This data-driven, causal framework revealed that post-lesional aggression does not stem from a monolithic locus, but is rigorously dictated by two distinct, reproducible network injury patterns that reconcile focal neuroanatomy with macro-scale network nomenclature (Miller et al., 2026). The first pattern is a frontomedial network pattern, centered on injuries to the vmPFC and ACC, which exhibits high functional connectivity with the SN. Disruption of this network essentially breaks the brain’s dynamic switchboard, causing a failure of top-down cognitive control and response inhibition that directly drives emotional disinhibition and explosive, impulsive conduct, providing the neurobiological architecture for “acquired sociopathy” (Box 2). Conversely, the second pattern is a temporo-amygdalar network pattern, localized within the medial temporal lobe, the amygdala, and the lateral OFC, which is functionally tied to the DMN and regions dedicated to social cognition. When this temporo-amygdalar network is compromised, the injury does not simply impair behavioral braking systems but fundamentally distorts real-time social information processing. According to integrative models of moral development, such as the Social Information Processing-Moral Decision-Making framework, the structural and functional uncoupling of this circuit restricts the cognitive resources required to objectively encode social contexts, leaving the patient unable to suppress internal hyper-vigilant states (Garrigan et al., 2018). Therefore, the persistent, un-decoupled DMN activity projects an implicit expectation of hostility onto the environment, misinterpreting neutral or ambiguous social cues as immediate, personal threats. This precise network entrapment generates the Hostile Attribution Bias (Box 1), transforming an objective processing failure into an unwarranted, defensive aggressive reaction. The dual-network causal architecture is further validated and generalized on a macroscopic scale by the meta-analysis conducted by Chen et al. (Chen et al., 2025), which maps these topographic alterations within an idiopathic, non-traumatic population explicitly screened to exclude individuals with a personal history of head trauma or a first-degree familiar history of psychosis. By synthesizing neuroimaging coordinates from 91 independent structural and functional studies involving thousands of aggressive individuals, the authors mapped the statistical convergence of gray matter volume, resting-state activity, and task-induced activations onto canonical large-scale networks. Intriguingly, the meta-analytic findings demonstrate a striking topographical convergence with the post-lesional data identified by Miller et al. (Miller et al., 2026). According to this global data synthesis, stability in aggressive traits is structurally rooted in significant gray matter volume reductions within the insula, superior temporal gyrus, and cingulate cortex, overlapping precisely with the core hubs of the SN. Furthermore, functional abnormalities during resting-state activity were localized within the mPFC and PCC, heavily implicating the DMN, while task-induced activations under provocation mapped directly onto the frontoparietal control system, or CEN. By contrasting these distinct methodologies, a crucial neuroforensic insight emerges, while Chen et al. (Chen et al., 2025) delineate the chronic network abnormalities typical of idiopathic developmental aggression, the causal lesion mapping of Miller et al. (Miller et al., 2026) proves that mechanical trauma, such as TBI, acts as a direct physical disruptor of these exact same neural scaffolds. However, while resting-state LNM establishes intrinsic connectivity constraints, it inherently lacks the resolution to capture how these lesions disrupt real-time cognitive and affective processing during active environmental challenges. To resolve this methodological barrier, a landmark meta-analytic connectivity (MACM) study by Dugré and Potvin mapped the task-based co-activation networks, of antisocial-onset lesions against a massive functional database of antisocial individuals (Dugré and Potvin, 2022). Their data-driven framework corroborates the network architecture by demonstrating that while temporal and amygdalar lesions selectively degrade the neural architecture required for emotional face evaluation and threat detection, orbitofrontal and frontomedial lesions directly impair reward-guided decision-making and gain evaluation. Using receptor density mapping, they revealed that the temporo-amygdalar processing failures are strictly bound to serotonergic dysregulation, whereas frontomedial deficits structurally decouple the endogenous opioid and dopaminergic pathways (Dugré and Potvin, 2022). As a further demonstration, the work of Peng et al. (Peng et al., 2025) maps lesion-related human aggression directly within a massive cohort of individuals with TBI. Specifically, the authors analyzed 182 veterans from the Vietnam Head Injury Study who sustained penetrating head injuries. Utilizing LNM, Peng et al. demonstrate that while the physical shrapnel or missile wounds are anatomically heterogeneous across the cohort, the injuries causally driving elevated aggression scores converge onto a single, universal functional network (Peng et al., 2025). Remarkably, this post-traumatic aggression network is defined by its strict connectivity to a distinct, localized “hub” region in the right dlPFC. This network exhibits strong positive connectivity with key areas including the vmPFC, the anterior insula, and the superior temporal gyrus, that perfectly align with the structural and functional coordinates derived by both Miller (Miller et al., 2026) and Chen (Chen et al., 2025). The dlPFC and temporal cortices may be critical not only for the expression of aggressive behavior, but for the modulation of implicit attitudes toward violence itself. A study including 112 male combat veterans with penetrating brain injuries, assessed approximately 40 years after injury - and 33 healthy controls - found that lesions involving the dlPFC were associated with stronger implicit violent attitudes (Cristofori et al., 2016). Moreover, deficits in executive functioning partially mediated this relationship, suggesting that the dlPFC contributes to the regulation of automatic responses toward violence through cognitive control mechanisms (Cristofori et al., 2016). Taken together, this evidence establishes that post-traumatic aggression can arise from mechanical disruption to a consistent, reproducible neural system, rather than an unpredictable psychiatric anomaly. In addition, mild TBI chronically impairs functional connectome dynamics, specifically degrading the fluid, real-time transitions between these exact same grand networks (DMN, SN, and CEN) in a strict age- and sex-dependent manner that features a premature loss of network flexibility in female patients and a relative early-adulthood resilience followed by a precipitously severe decline in network fluidness during advanced senescence in male patients (Amgalan et al., 2022).

Structural connectivity studies further suggest that these functional abnormalities are rooted in DAI. DTI studies consistently demonstrate reduced white matter microstructural integrity following TBI (Shenton et al., 2012; Hulkower et al., 2013), which compromises communication between distributed neural systems (Hulkower et al., 2013) and disrupts large-scale network integration (Hayes et al., 2016). In particular, patients with chronic mild TBI and elevated aggression scores exhibit increased radial diffusivity within the corpus callosum, indicating reduced white matter integrity and impaired interhemispheric communication (Dailey et al., 2018), as further demonstrated by preclinical evidence (Song et al., 2005). Crucially, this post-traumatic structural deafferentation mirrors broader neuroforensic models of affective dysregulation, which demonstrate that reactive aggression is fundamentally predicated on an objective hyper-reactivity and hypersensitivity of the amygdalar core to perceived threats, a phenotype that stands in stark contrast to the amygdala hypo-reactivity observed in developmental psychopathy and proactive, instrumental violence (Tonnaer et al., 2023). From a neurodevelopmental perspective, the construction of this neuromoral network follows an asynchronous trajectory: while the amygdala matures early in life to serve as an emotional anchor for empathy and moral learning, the prefrontal structures undergo a protracted myelination continuing into early adulthood to provide the cognitive architecture for behavioral governance (Tonnaer et al., 2023). When TBI disrupts this delicately integrated system, it functionally untethers the early-maturing emotional signaling from its late-maturing prefrontal brakes, leading to a profound significant impairment of the neural substrates that subserve moral decision-making and social conduct (Tonnaer et al., 2023).

The amygdala-centered dysfunction varies by subtype: reactive aggression is typically marked by amygdala hyper-responsivity, whereas proactive or psychopathic aggression is associated with reduced amygdala volume and emotional blunting (Pardini et al., 2014) (Box 2). In line with this framework, evidence from 49 male veterans indicates that mild TBI, together with post-traumatic stress disorder (PTSD) and related neuropsychiatric comorbidities, is associated with increased risk of intimate partner violence perpetration, in both its physical and psychological form. Notably, aggression severity has been linked to microstructural alterations within limbic circuits, including the amygdala-hippocampal complex, supporting the notion that TBI-related disruptions of amygdala-centered networks involved in threat processing and affect regulation may contribute to violent behavior (Rojczyk et al., 2024).

As described in Section 3, the TBI-related impairment of white matter pathways and functional network integrity are accompanied by widespread perturbations of neurotransmitter systems. Such perturbations further compromise communication between distributed brain regions and contribute to the emergence of post-traumatic behavioral disturbances, including aggression.

TBI induces a severe alteration of cholinergic neurotransmission. The behavioral consequences of this cholinergic failure, however, extend beyond the disruption of oscillatory synchrony: the post-traumatic loss of ACh-mediated gain control removes a critical filter that distinguishes high-priority threats from irrelevant provocations, and in doing so, lowers the threshold for reactive aggression while dismantling the circuit-level capacity for its inhibition. The link between cholinergic signaling and the regulation of aggression is supported by converging evidence across levels of analysis. At the genetic level, acetylcholinesterase (ACHE), the primary enzyme responsible for ACh degradation, have been identified among the top genetic correlates of trait aggression in large-scale human studies, suggesting that basal cholinergic tone exerts a tonic modulatory influence on aggressive propensity (Zhang-James et al., 2019). At the receptor level, nicotinic acetylcholine receptors (nAChRs), and particularly the α7 subunit, constitute a consistent pharmacological substrate for aggression modulation: nAChR agonists reduce offensive, defensive, and predatory aggression across species and reduce aggression-related impulsivity in human laboratory paradigms (Lewis and Picciotto, 2020). The unifying mechanism, as described in Section 2, is that α7 nAChRs on late-firing GABAergic interneurons of the BLA provide a tonic inhibitory brake on principal neuron output; their activation suppresses amygdala reactivity to aversive stimuli, while their TBI-induced loss disinhibits BLA principal neurons, reconfiguring the amygdala toward a hyperexcitable state in which the signal-to-noise sharpening described in the healthy brain is replaced by indiscriminate, amplified reactivity to provocative environmental triggers (Lewis and Picciotto, 2020; Pidoplichko et al., 2013). This post-traumatic disinhibition carries direct forensic significance. The cholinergic regulation of impulsivity, mediated through prefrontal ACh release and its modulation of attentional filtering and response inhibition, is selectively eroded by the NBM degeneration that characterizes chronic TBI (Titus et al., 2019). Stripped of the prefrontal cholinergic tone that normally sustains the capacity to evaluate the consequences of a behavioral response before its execution, the injured brain loses a primary neurochemical mechanism for aborting reactive aggression once the amygdala has been engaged. Crucially, this failure is not redundant with the dopaminergic and serotonergic deficits described below: whereas post-traumatic dopaminergic hypofunction undermines reward-guided behavioral valuation and serotonergic depletion blunts prefrontal inhibitory efficacy at the circuit level, the cholinergic deficit operates upstream, impairing the attentional and oscillatory infrastructure that determines which stimuli are assigned threat salience in the first place. In this sense, by degrading the BLA’s capacity to discriminate between genuine and spurious threats, post-traumatic ACh failure systematically expands the range of stimuli capable of recruiting the dopaminergic, serotonergic, and noradrenergic pathways that mediate reactive aggressive output, amplifying the neurobiological vulnerability profile described throughout this section.

Within the framework of post-traumatic and antisocial aggression, the dopaminergic pathways emerge as modulators, governing behavioral control, reward processing, and resilience to frustration. Although scientific literature specifically focused on TBI rarely analyzes the direct link between brain damage, dopamine, and antisocial conduct simultaneously, a body of neurobiological and neuroimaging evidence on aggressive and violent offenders allows us to map the vulnerability profile associated with dopaminergic dysfunction. [^18^F]-fluoro-L-DOPA PET studies demonstrated that a reduced capacity for dopamine synthesis and storage at the subcortical level significantly correlates with a greater propensity to react aggressively and punitively (Schlüter et al., 2013). Individuals with deficits in baseline dopaminergic transmission exhibit lower cognitive resilience and a marked inability to inhibit reactive impulses, preferring immediate retaliation over goal-directed behavioral maintenance. This finding suggests that dopaminergic hypofunction acts as a neurobiological vulnerability factor for impulsive aggression. The architecture of dopamine D1-and D2-receptors modulates the balance between prosocial and antisocial behaviors, as reduced D1-receptor availability in the limbic striatum is associated with higher levels of trait physical aggression in healthy human subjects (Plavén-Sigray et al., 2014). Concurrently, the D2-receptor system appears profoundly altered in more severe criminal and antisocial manifestations and in violent offenders with high psychopathic traits (Lukkarinen et al., 2024). Within this population, the reduction of D2-receptor binding in the ventral striatum and the amygdala inversely correlates with the severity of antisocial and psychopathic symptomatology. This form of baseline dopaminergic blunting may drive individuals toward pathological sensation-seeking and instrumental, appetitive antisocial acts to engage a chronically under-responsive reward system. This perspective is further supported by clinical and forensic frameworks, demonstrating that dysregulations within dopaminergic circuits distort reward processing and behavioral valuation (Sarkar and Wrangham, 2023). This receptor-driven valuation deficit is structurally and functionally mirrored in the lesion-mapping literature. When analyzing brain injuries that causally trigger antisocial behavior, task-based meta-analytic co-activation modeling isolates a distinct frontomedial and orbitofrontal network that selectively disrupts reward-guided decision-making and gain evaluation, demonstrating a strong spatial correlation with subcortical dopaminergic receptor profiles (Dugré and Potvin, 2022). According to this model, an altered dopamine system compromises the top-down inhibitory control exerted by the prefrontal cortex over subcortical structures. The neurochemical imbalance biases the individual toward immediate gratification and short-term reward pursuit, severely undermining the ability to foresee the negative consequences of violent actions and drastically inflating the risk of antisocial and aggressive outbursts. Crucially, the loss of top-down regulation operates in parallel with maladaptive synaptic remodeling within subcortical aggressive cores triggered by traumatic experiences. Preclinical evidence demonstrates that traumatic stress induces a robust, long-lasting LTP at key subcortical synapses, specifically reinforcing the pathways running from the MeA to the hypothalamus and the BNST (Nordman et al., 2020). This traumatic stress-induced synaptic potentiation persists for at least 1 week and selectively drives a prolonged increase in offensive aggression, characterized by elevated attack time, increased frequency of bouts, and drastically reduced attack latency, while leaving non-aggressive social communication and locomotion unaltered (Nordman et al., 2020). Post-traumatic or injury-induced hyper-potentiation locks the MeA-Hypothalamus and MeA-BNST circuits into a hyperexcitable configuration, lowering the threshold for unprovoked escalations (Nordman et al., 2020). In summary, the integrated data indicates that both presynaptic deficits in dopamine synthesis and alterations in post-synaptic D1 and D2 receptor density within the limbic system deprive the brain of the necessary mechanisms for emotional regulation and delayed gratification. Ultimately, within the complex sequelae of TBI, the dopaminergic hypofunction and reward dysregulation carries profound clinical significance as a primary causal factor behind the onset of post-traumatic emotional dysregulation and violence.

Building upon the structural dismantling of ascending DRN projections described in Section 3, the chronic neurochemical landscape of post-traumatic serotonergic failure evolves beyond acute depletion into a maladaptive neuroadaptive state that durably reconfigures corticolimbic reactivity. Individuals exhibiting aggressive behaviors demonstrate altered neuromodulation of the above-described circuits. In this domain, serotonergic dysfunction is considered one of the principal mechanisms underlying impulsive aggression and violence (Box 2) (Volavka, 2002). Chronic deficits in 5-HT neurotransmission severely blunt the inhibitory efficacy of the prefrontal brake, directly correlating with the emergence of impulsive, reactive, and affective aggression and increasing the sensitivity to perceived threat (Siever, 2008; Cupaioli et al., 2021). The biochemical foundation of this relationship is provided by the seminal forensic evidence of Virkkunen et al. (Virkkunen et al., 1995), who demonstrated that impulsive violent offenders, including those meeting criteria for antisocial personality disorder (ASPD) and intermittent explosive disorder (IED) (Box 2), exhibit significantly lower cerebrospinal fluid (CSF) 5-HIAA concentrations compared to non-impulsive offenders, with this neurochemical deficit prospectively predicting recidivist violent behavior following release (Virkkunen et al., 1995). Critically, clinical evidence directly linking this neurochemical deficit to behavioral dysregulation in TBI populations is provided by the observation that patients sustaining frontal contusions exhibit significantly lower CSF 5-HIAA concentrations compared to TBI patients without aggressive behavior, with selective serotonin reuptake inhibitors (SSRIs) administration concurrently ameliorating both depressive and aggressive symptomatology (Jorge and Robinson, 2003). The stability in aggressive traits is directly rooted in a severe, compensatory upregulation and hypersensitivity of post-synaptic 5-HT receptors within the prefrontal structures that fails to restore homeostasis (Coccaro, 1989). Refining this framework, modern *in vivo* molecular imaging demonstrates that this homeostatic failure is driven by chronically low interstitial 5-HT levels, critically indexed by elevated serotonin transporter (SERT or 5-HTT) density and a compensatory upregulation of frontolimbic 5-HT receptors (da Cunha-Bang and Knudsen, 2021). As established in Section 2, the receptor-network architecture delineated by Salvan et al. (Salvan et al., 2023) specifies precisely which serotonergic substrate is dismantled by this neurochemical deficit: the first mode of serotonergic population variation - anchored in the 5-HT_1A_, 5-HT_1F_, and 5-HT_2C_ networks within the amygdala and temporal cortex, and physiologically constituted by the convergent Gi/o-mediated and Gq-mediated inhibitory mechanisms described above - is selectively deprived of its ligand (Salvan et al., 2023). Post-traumatic structural destruction of DRN projections, combined with the compensatory but functionally inadequate upregulation of these receptor populations, persistently impairs this modulatory mode, depriving the amygdala and temporal cortex of the serotonergic signal that physiologically gates reactive aggression and antisocial conduct. The receptor-network deficit is causally validated by task-based lesion network mapping, which demonstrates that heterogeneous injuries localized within the medial temporal lobe and amygdala consistently converge onto aberrant co-activation networks dedicated to emotional face processing and threat detection, with these processing failures being strictly and selectively bound to underlying SERT and 5-HT_1A_ receptor density maps (Dugré and Potvin, 2022). This mechanistic specificity transforms the post-traumatic neurochemical depletion from a generic monoaminergic deficit into a targeted disruption of the receptor-network substrate that physiologically subserves behavioral inhibition and social conduct, inducing a profound functional uncoupling within corticolimbic networks and rendering subcortical nodes like the amygdala and dorsal striatum hyper-reactive to provocative environmental triggers (da Cunha-Bang and Knudsen, 2021), precisely replicating, through a structural rather than modulatory mechanism, the corticolimbic dysregulation that defines primary impulsive aggression syndromes.

The role of NE in aggressive behaviors comes into play, revealing that its involvement is not primary, but rather secondary and strictly dependent on the stress-response system (Haden and Scarpa, 2007). Rather than originating the aggressive impulse itself, NE acts as a neuromodulator that primes the organism for action upon perceiving an environmental threat. Crucially, this secondary involvement manifests differently depending on the sub-type of violence expressed, drawing a sharp line between impulsive/reactive aggression and instrumental/proactive aggression (Haden and Scarpa, 2007) (Box 2). Impulsive, reactive, or affective aggression, characterized by a sudden loss of behavioral control and high emotional turbulence, is strongly associated with a hyper-reactive noradrenergic state and elevated autonomic arousal in both nonhuman animals and humans (Haden and Scarpa, 2007). In this scenario, when the LC-Amygdala axis is overactivated by stress, the massive release of NE in the BLA amplifies emotional reactivity and fear, lowering the threshold for violent outbursts. Preclinical evidence from animal models indicates that this noradrenergic hyper-arousal not only cements aversive memories but can also impair the extinction of fear, locking the organism into a state of persistent hyper-vigilance where neutral stimuli are continuously parsed as imminent threats (Giustino and Maren, 2018). Here, aggression represents an extreme, dysregulated behavioral manifestation of the fight-or-flight response (Buffalari and Grace, 2007; McCall et al., 2017; Haden and Scarpa, 2007). Conversely, instrumental, proactive, or predatory aggression, which is cold, calculated, premeditated, and detached from emotional triggering, displays a completely different neurobiological profile, often correlating with a hypo-reactive noradrenergic system and blunted autonomic responses (Haden and Scarpa, 2007). Individuals engaging in instrumental violence frequently exhibit a lack of physiological fear and emotional empathy, conditions sustained by a hypo-functioning LC-NE axis that fails to recruit the amygdala during aversive challenges (Haden and Scarpa, 2007). Therefore, the involvement of NE in aggression cannot be generalized: it is a secondary, vicarious phenomenon where NE acts as a catalyst that drives reactive, stress-induced violence while remaining strikingly absent or diminished in predatory, cold-blooded aggression.

Collectively, these findings support a multilevel neurobiological framework in which structural lesions, network dysconnectivity, limbic hyper-reactivity, and neurotransmitter imbalance converge to drive the multifaceted expressions of post-traumatic aggressive and antisocial conduct. This complex biological matrix introduces profound challenges when transitioned from the laboratory to the courtroom, where concepts of free will, blameworthiness, and behavioral control must be legally quantified (see Box 3). A summary of the human and preclinical evidence supporting the major proposed neurobiological mechanisms of post-traumatic aggression is presented in Table 1.

Box 3 **Neuro-forensic and legal translation.**

**Methodological note for the reader**

The translation of neurobiological data into the legal arena requires mapping structural brain abnormalities onto specific statutory definitions of criminal responsibility, cognitive capacity, and volitional control. In forensic settings, proving an organic deficit is insufficient; a rigorous causal nexus must be established between network dysfunction and the specific criminal act.

**The architecture of criminal responsibility**

• **The Dichotomy of Imputability (Mens Rea vs. Actus Reus):** In most international legal systems, criminal culpability requires the coexistence of the physical act (actus reus) and a guilty, intentional mind (mens rea). Severe post-traumatic frontolimbic disconnection directly challenges the mens rea requirement. While the physical act is executed, the internal capacity to formulate genuine, normative legal intent or to fully comprehend the moral weight of the action is compromised by the underlying organic disruption.
• **Cognitive Capacity vs. Volitional Control:** A crucial legal distinction illuminated by traumatic brain injury (TBI). Patients suffering from post-traumatic Acquired Sociopathy or PCDAMC frequently retain intact cognitive capacity (the abstract, intellectual knowledge of right from wrong, often mediated by a spared dorsolateral prefrontal cortex). However, they systematically lose volitional control (the neurobiological capacity to inhibit an action and modulate behavior in accordance with that knowledge, due to a disrupted ventromedial and orbitofrontal cortex).
• **The Determinism Dilemma (The 'Bad' vs. 'Mad' Question):** When a mechanical trauma effectively dismantles the neural substrates of behavioral containment, it forces a profound reconsideration of the classical boundaries of free will. This neuro-legal intersection leaves open a critical, yet unresolved question: to what extent can an organically compromised individual be deemed legally blameworthy if their criminal conduct arises from a biophysically predetermined failure of inhibitory control, rather than a conscious, malevolent choice?

**Forensic defense standards and casual nexus**

• **The “Irresistible Impulse” Doctrine:** This legal standard is highly applicable to post-traumatic reactive aggression. It argues that even if the defendant intellectually understood that the act was illegal (intact cognition), a profound structural failure of top-down cortical braking systems (OFC/vmPFC) rendered them organically incapable of resisting the impulsive, bottom-up urge generated by the hyper-reactive limbic core. The action becomes an unmodulated biological reflex rather than a conscious choice.
• **Act-Specific Neuro-Causal Nexus:** To be legally admissible, forensic evaluation must move beyond static imaging findings. Discovering a brain abnormality (e.g., an incidental focal lesion or diffuse axonal injury via DTI) is legally meaningless unless the defense expert establishes a strict, direct causal link proving that this specific lesion directly mediated the behavioral disinhibition (impulsivity or irritability) at the exact millisecond the crime was committed.

**Post-trial and prognostic assessments**

• **Future Dangerousness and Risk Assessment:** In forensic and penitentiary settings, structural frontolimbic damage combined with a chronically dysregulated Salience Network creates a complex prognostic profile. While these organic deficits elevate the baseline risk of violent recidivism, utilizing neuroimaging to predict “future dangerousness” remains highly controversial in legal theory, as it risks penalizing an individual based on a deterministic, static structural deficit rather than active behavioral choices or potential rehabilitative progress.

**TABLE 1:** Evidence supporting proposed neurobiological mechanisms of post-traumatic aggression.

Major proposed mechanism	Source of evidence (human/preclinical)	Techniques used	References
Large-scale network disruption (frontomedial and temporo-amygdalar networks)	Human (TBI cohorts, violent offenders, antisocial individuals, healthy controls)	Lesion network mapping (LNM); structural and functional neuroimaging; meta-analytic connectivity modeling (MACM)	(Miller et al., 2026; Chen et al., 2025; Dugré and Potvin, 2022; Peng et al., 2025; Amgalan et al., 2022)
Diffuse Axonal Injury (DAI) and Structural Deafferentation	Human (Mild TBI) and Preclinical	Diffusion tensor imaging (DTI); radial diffusivity; immunostaining	(Dailey et al., 2018; Shenton et al., 2012; Hulkower et al., 2013; Song et al., 2005)
Cholinergic alterations and Threat salience	Human and Preclinical	Genome-wide association studies (GWAS) and transcriptome analyses; receptor binding assays, pharmacological challenge paradigms, and electrophysiology	(Zhang-James et al., 2019; Lewis and Picciotto, 2020; Titus et al., 2019)
Dopaminergic dysregulation and Reward valuation	Human (violent offenders, healthy subjects) and Preclinical	$\left[18F\right]$ -fluoro-L-DOPA PET, Radioligand PET binding (D1/D2 receptors), Meta-Analytic Connectivity Modeling (MACM) and receptor density mapping, Brain slice electrophysiology	(Dugré and Potvin, 2022; Schlüter et al., 2013; Plavén-Sigray et al., 2014; Lukkarinen et al., 2024)
Serotonergic disruptions and Corticolimbic disinhibition	Human (Impulsive violent offenders, TBI patients) and Preclinical	Transcriptomic-neuroimaging mapping; optogenetics-functional magnetic resonance imaging (MRI) and pharmacology; Meta-Analytic Connectivity Modeling (MACM) and receptor density mapping Cerebrospinal fluid (CSF) 5-HIAA quantification and pharmacological intervention (SSRIs); Molecular PET imaging	(Salvan et al., 2023; Dugré and Potvin, 2022; Virkkunen et al., 1995; Jorge and Robinson, 2003; da Cunha-Bang and Knudsen, 2021)
Noradrenergic hyper-arousal (reactive)/hypo-arousal (proactive aggression)	Human and Preclinical	Autonomic reactivity and psychophysiology (heart rate, skin conductance), Task-based fMRI; Retrograde tracing, fast-scan cyclic voltammetry, electrophysiology, and *in vivo* optogenetics	(McCall et al., 2017; Haden and Scarpa, 2007)

## 5 Pharmacological therapies

Given the marked clinical and neurobiological heterogeneity of TBI, current management strategies emphasize a multidisciplinary and individualized approach integrating neuropsychiatric assessment, environmental modulation, behavioral rehabilitation, pharmacological and/or neuromodulation intervention. Pharmacological interventions, described as effective in the treatment of neuropsychiatric complications following TBI, act on neurochemical systems diffusely and non-topographically, often leaving the dysregulated frontolimbic connectivity incompletely addressed. Non-pharmacological treatments, including psychotherapy (Wiart et al., 2012), such as cognitive behavioral therapy (CBT) (Iruthayarajah et al., 2018), behavioral modification programs, relaxation-based interventions, and social skills training, have demonstrated variable but clinically meaningful benefits, particularly in reducing emotional dysregulation and improving adaptive coping strategies (Alderman, 2003). Due to its structured and goal-directed nature, CBT and associated psychoeducational protocols are particularly well-suited for managing post-traumatic anger and aggressiveness, as they allow clinicians to bypass underlying executive dysfunction through targeted cognitive rehabilitation strategies (Coetzer, 2009; Kangas and McDonald, 2011; Jeffay et al., 2023). Indeed, adapted cognitive rehabilitation programs emphasizing self-awareness, reasoning skills, and tailored self-management strategies have demonstrated feasibility and initial efficacy in mitigating neurobehavioral sequelae in chronic TBI populations (Aboulafia-Brakha et al., 2013; Hart et al., 2015). Nevertheless, pharmacological treatment remains widely employed in moderate-to-severe cases, although evidence-based prescribing practices remain limited and heterogeneous. Here we will briefly review the state-of-the art of main pharmacology approaches applied to the most prevalent and disabling neurobehavioral sequelae of TBI.

Aggression represents one of the most prevalent and disabling neurobehavioral sequelae of TBI, manifesting as verbal hostility, impulsive outbursts, physical violence, or destructive behavior, with substantial repercussions on social functioning, rehabilitation adherence, interpersonal relationships, and community reintegration (Hicks et al., 2019; Bales et al., 2009). Current literature provides the strongest support for amantadine and beta-adrenergic blockers, both of which have shown efficacy in reducing irritability and aggressive behavior following TBI (Hicks et al., 2019; Pisa et al., 2015; Mehta et al., 2018). Amantadine is thought to exert its therapeutic effects through modulation of dopaminergic transmission and enhancement of frontal network activity, whereas beta-blockers may attenuate aggression by reducing adrenergic hyperarousal and autonomic reactivity.

Additional pharmacological approaches have targeted the principal neurochemical systems implicated in post-traumatic aggression, including serotonergic, dopaminergic, and catecholaminergic pathways (Hicks et al., 2019; Bales et al., 2009; Yue et al., 2017). Anticonvulsants such as carbamazepine and valproate have been investigated for their mood-stabilizing and anti-impulsive properties, although results remain mixed. In a randomized double-blind placebo-controlled trial, carbamazepine demonstrated signals of clinical benefit despite substantial placebo effects, supporting its continued use in selected patients under specialist supervision (Hammond et al., 2021). Similarly, SSRIs, tricyclic antidepressants, lithium, methylphenidate, and atypical antipsychotics have been explored as adjunctive treatments, whereas typical antipsychotics should generally be used cautiously because of their potential adverse cognitive and neurological effects (Luauté et al., 2016; Plantier et al., 2016). Lithium, a medication that appears to reduce suicide risk for bipolar patients, also reduced impulsive aggressive behavior in prisoners who were not psychotic; both effects could be mediated by an enhancement of serotonergic activity (Sheard et al., 1976). A case report shows neuropsychiatric sequelae of TBI responding well to lithium and animal studies suggest that pre-injury lithium treatment may improve neurocognitive recovery, but with no clinical or dosing guideline yet established, underscoring the need for controlled trials before lithium can be recommended for psychiatric sequelae of TBI (Leeds et al., 2014; Aswani et al., 2022).

Among atypical antipsychotics, risperidone has attracted interest because of its combined serotonergic and dopaminergic receptor antagonism, potentially relevant for regulating impulsivity and affective dysregulation after TBI. In a randomized controlled trial comparing risperidone with placebo for post-traumatic aggression, Deb et al. demonstrated that recruitment and retention of this patient population into blinded pharmacological trials was achievable, although definitive efficacy conclusions were limited by sample size and methodological constraints. Nonetheless, the study highlighted the ongoing clinical use of risperidone in managing severe aggression and irritability following TBI, while underscoring the need for larger adequately powered trials to better define its efficacy, tolerability, and cognitive safety profile in this vulnerable population (Deb et al., 2020).

More recently, translational and preclinical studies have explored novel compounds capable of modulating frontolimbic circuitry. In an experimental model of mild TBI (mTBI), two-pentadecyl-2-oxazoline (PEA-OXA) significantly attenuated both behavioral and neuropsychiatric alterations following mTBI. The compound was shown to act as an α2-adrenergic antagonist. In mTBI mice, anxiety-like and aggressive behaviors emerged 14 days post-injury, whereas depressive-like symptoms and social deficits developed from day 60 onward. PEA-OXA normalized these behavioral abnormalities and, following microinjection into the medial prefrontal cortex, enhanced dopamine release. Overall, PEA-OXA exerts multimodal neurobiological effects, including improvement of behavioral dysfunction, normalization of cortical GABAergic activity, and restoration of impaired prefrontal neuronal function (Boccella et al., 2020). These findings are particularly relevant given the growing evidence that post-traumatic aggression arises from impaired prefrontal inhibitory control over hyper-reactive limbic networks.

Taken together, these findings underscore the marked heterogeneity of post-traumatic aggression, which is shaped by differences in injury severity, and the specific neural circuits involved, as well as by the broad phenotypic variability of aggressive manifestations. A more comprehensive characterization of these interacting determinants may ultimately enhance the prediction of long-term recovery trajectories and functional outcomes following traumatic brain injury. Given at a sufficiently high dose, many drugs will temporarily reduce or eliminate violent or aggressive behavior. High doses of hypnotics, antipsychotics, anticonvulsants, or general anesthetics will make it difficult or impossible to exhibit violent behavior. Such doses will also make it difficult or impossible to exhibit any other behavior; the effect is non-specific sedation (Volavka, 2002). Overall, advancing the field will require a precision-medicine framework capable of capturing the dynamic interplay between lesion-related neurobiology, individual predispositions, and environmental influences, thereby providing a more robust basis for tailoring integrated interventions and identifying biomarkers predictive of persistent aggression and long-term psychosocial adaptation after TBI.

## 6 Neuromodulation therapies

Neuromodulation techniques offer a mechanistically complementary strategy: rather than addressing neurotransmitter availability, they target the spatial and temporal architecture of dysfunctional circuits, aiming to restore oscillatory coherence, excitation-inhibition balance, and effective prefrontal-to-amygdala communication (Rabinowitz and Levin, 2014; Cicerone et al., 2019; Pevzner et al., 2016). A crucial advantage in patients with head trauma is the ability to personalize therapy based on individual neuroradiological and electrophysiological profiles: a precision medicine approach that considers the heterogeneity of lesions, an intrinsic characteristic of this population.

Repetitive transcranial magnetic stimulation (rTMS) modulates cortical excitability beyond the stimulation period: high-frequency protocols (≥10 Hz) are generally facilitatory, low-frequency protocols (≤1 Hz) suppressive (Fitzgerald et al., 2006; Hallett, 2007). The most relevant targets for post-traumatic frontolimbic dysregulation are the left dlPFC, to restore top-down inhibition to the amygdala *via* the CEN, and the mPFC/OFC, to functionally compensate for the inhibitory projection from the vmPFC to the amygdala disrupted by damage to the uncinate fasciculus (Fitzgerald et al., 2006; Sacco et al., 2016). Theta-burst stimulation (TBS) is particularly compelling: intermittent TBS over the left dlPFC can be conceptualized as an exogenous restoration of the theta rhythmogenesis that TBI disrupts, given that the BLA relies on NBM-driven theta oscillations to maintain LTP-permissive windows and frontolimbic coupling (Huang et al., 2005).

Transcranial direct current stimulation (tDCS) delivers a weak direct current (1–2 mA) that produces polarity-dependent changes in resting membrane potential, without directly inducing action potentials, with effects that persist even after stimulation due to NMDA receptor-dependent plasticity (Nitsche and Paulus, 2000). Anodal stimulation of the left dlPFC or vmPFC, paired with cathodal placement over right supraorbital regions, reduced reactive aggression behavior in controlled paradigms (Dambacher et al., 2015). In TBI populations, variability in white matter integrity and cortical thickness critically determines target current density; therefore, individualized computational modeling of current flow using patient-specific MRI is increasingly recommended (Bikson et al., 2016).

Neurofeedback enables operant conditioning of specific oscillatory patterns through real-time feedback of the patient’s own neural activity (Marzbani et al., 2016). Since post-traumatic frontolimbic dysregulation is characterized electrophysiologically by the loss of frontal theta coherence and the pathological predominance of slow waves (Horvat et al., 2023; Stevens et al., 2024), protocols targeting frontal theta enhancement or alpha-band asymmetry normalization are mechanistically grounded. Studies in patients with traumatic brain injury and persistent affective symptoms have found that neurofeedback produced significant reductions in emotional dysregulation and frequency of aggression, with improvements correlating with post-treatment increases in frontal theta coherence (Bennett et al., 2018; Chen et al., 2023).

Transcranial Focused Ultrasound (tFUS) delivers low-intensity pulsed ultrasound to a target of approximately 3–5 mm resolution, modulating mechanosensitive ion channels without tissue damage (Jin et al., 2024). Its key advantage is the capacity to reach deep subcortical structures non-invasively: amygdala, thalamus, and hippocampus all lie within effective range. It has been demonstrated that tFUS applied to the amygdala reduces BOLD responses to threatening stimuli and attenuates physiological arousal (Barksdale et al., 2025). The ability to selectively target BLA *versus* CeA based on stereotactic neuronavigation represents a qualitative advance over surface-based techniques, aligning therapeutic precision with the nuclear-resolution circuit anatomy described in this review.

Vagus nerve stimulation (VNS) modulates frontolimbic circuits indirectly by activating the nucleus of the solitary tract and, *via* the LC, driving widespread noradrenergic and cholinergic projections to cortical and subcortical targets (Howland, 2014; Huang et al., 2023; Yakunina et al., 2017). The transcutaneous auricular form (taVNS) achieves comparable central effects through non-invasive ear-clip stimulation. In the context of post-traumatic aggression, taVNS is directly relevant: it can partially compensate for the LC-amygdala noradrenergic dysregulation described above, attenuate the chronic neuroinflammatory cascade that sustains PV^+^ interneuron apoptosis, and can partially restore NBM-dependent theta rhythmogenesis. (Broncel et al., 2018; Kraus et al., 2007; Song et al., 2025).

Deep brain stimulation (DBS) delivers continuous high-frequency electrical stimulation to stereotaxically implanted subcortical electrodes and remains a clinically validated option for severe, pharmacologically refractory behavioral dysregulation (Pycroft et al., 2018). Relevant DBS targets for posttraumatic aggression include the posterior hypothalamus, as a downstream effector of medial amygdala-driven aggressive behavior (Contreras Lopez et al., 2021; Franzini et al., 2005) and the BNST, a key node in the medial amygdala output pathway that regulates the initiation of aggression (Lebow and Chen, 2016; Lischinsky and Lin, 2020). BLA-targeted DBS has also been reported in isolated cases of severe self-aggressive behavior (self-injurious behavior in autism) with observed behavioral normalization, though evidence remains limited to uncontrolled series (Benedetti-Isaac et al., 2023). A key ethical dimension distinguishes DBS from all other interventions reviewed here: the direct modulation of circuits mediating emotional experience and volitional control in patients whose decision-making capacity is already organically compromised requires rigorous multidisciplinary ethical evaluation, with particular attention to the forensic implications outlined in Box 3.

All approaches above share a fundamental limitation: pre-programmed parameters are delivered regardless of the patient’s instantaneous neural state. Closed-loop neuromodulation addresses this by continuously monitoring a neural biomarker (such as amygdala high-gamma activity, frontal theta coherence, or local field potential power) and delivering corrective stimulation only when a pathological threshold is crossed (Sellers et al., 2023). This adaptive architecture is particularly relevant for post-traumatic aggression: if the pathological state is defined by amygdala hyperexcitability and theta desynchronization, a closed-loop system could detect electrophysiological precursors of an aggressive episode and trigger prefrontal or BNST stimulation before behavioral escalation occurs. Early closed-loop DBS systems for epilepsy and Parkinson’s disease have demonstrated that biomarker-driven adaptive stimulation outperforms open-loop continuous stimulation while reducing side effects (Wang et al., 2023; Wu YC. et al., 2021); translation to psychiatric applications is an active area of investigation.

Structured cognitive rehabilitation, such as attention training, CBT for emotional regulation, social cognition programs, operates as functional neuromodulation, strengthening prefrontal-to-limbic inhibitory connections through Hebbian plasticity (Fliti et al., 2025; Saccenti et al., 2024). Neuroimaging evidence shows that successful anger-management CBT in TBI is associated with measurable increases in dlPFC-amygdala functional connectivity and OFC normalization during emotional conflict tasks (Sandman et al., 2020). Combining rehabilitation with simultaneous tDCS and/or rTMS exploits the state-dependent nature of stimulation-induced plasticity, ensuring that increased cortical excitability coincides with active engagement of the target circuit (Kwon et al., 2016; Zhang et al., 2026). Rather than serving as empirical symptomatic treatments, modern neuromodulation modalities and pharmacological approaches can be mechanistically matched to the specific post-traumatic circuit defects identified *via* quantitative neuroimaging and electrophysiology. For instance, traumatic disruption of the uncinate fasciculus and NBM-derived cholinergic pathways leads to a loss of frontal theta coherence and unmodulated amygdalar hyper-reactivity (see Table 2). High-frequency rTMS or intermittent theta-burst stimulation (iTBS) delivered over the left dlPFC directly addresses this by exogenously driving cortical excitability and restoring theta-band phase-locking across frontolimbic networks (Fitzgerald et al., 2006; Sacco et al., 2016; Huang et al., 2005; Nitsche and Paulus, 2000; Dambacher et al., 2015; Bikson et al., 2016). To translate these approaches into current decision-making, patient selection should be guided by quantitative EEG metrics - specifically reduced frontal theta coherence and alpha asymmetry - which serve as physiological biomarkers of frontolimbic desynchronization. Similarly, for deep subcortical hubs like the basolateral amygdala or BNST, non-invasive tFUS offers nuclear-resolution targeting informed by patient-specific DTI tractography (Jin et al., 2024; Barksdale et al., 2025). The ultimate frontier lies in adaptive, closed-loop neuromodulation, where continuous sensing of electrophysiological biomarkers (such as amygdalar high-gamma power surges or theta desynchronization) triggers localized stimulation before affective dysregulation escalates into overt reactive aggression. A summary of behavioral manifestations with respective candidate therapeutical intervention(s) (pharmacological and neuromodulatory alike) for every circuit this work discusses, is proposed in Table 2.

**TABLE 2:** Major neural circuits, associated behavioral manifestations and candidate therapeutic interventions in post-traumatic aggression and antisocial behavior.

Neural circuit/system	Associated behavioral manifestation(s)	Biomarker/stratification Tool	Candidate therapeutic Intervention(s)
Large-scale fronto-limbic networks (DMN–SN–CEN triple network; uncinate fasciculus; vmPFC/ACC–amygdala decoupling)	Loss of top-down inhibitory control; hostile attribution bias; emotional disinhibition; “acquired sociopathy”/PCDAMC (Menon, 2023; Bonnelle et al., 2012; Von Der Heide et al., 2013; Miller et al., 2026; Garrigan et al., 2018)	Decreased frontal theta coherence (qEEG); Reduced fractional anisotropy in uncinate fasciculus (DTI)	*Neuromodulation – device-based:* rTMS/iTBS over left dlPFC and mPFC/OFC (Fitzgerald et al., 2006; Sacco et al., 2016; Huang et al., 2005); anodal tDCS over left dlPFC/vmPFC (Nitsche and Paulus, 2000; Dambacher et al., 2015; Bikson et al., 2016); *Neuromodulation – behavioral/functional:* CBT and structured cognitive rehabilitation (Fliti et al., 2025; Saccenti et al., 2024; Sandman et al., 2020; Kwon et al., 2016; Zhang et al., 2026).
BLA microcircuit (PV^+^ interneuron loss, E/I imbalance, dendritic hypertrophy, chronic neuroinflammation)	Reactive/impulsive aggression; amygdalar hyper-reactivity to threat; hyperarousal (Freund and Katona, 2007; McCorkle et al., 2021; Tonnaer et al., 2023; Pardini et al., 2014)	Local hyper-reactivity signals (fMRI/PET); Pathological gamma/theta power shifts	*Neuromodulation – device-based:* tFUS targeted to BLA; taVNS – anti-inflammatory, theta-restoring (Broncel et al., 2018; Song et al., 2025); *Pharmacological:* PEA-OXA – preclinical (Boccella et al., 2020).
Cholinergic system (NBM → BLA/amygdala; α7 nAChR, M1/M3 receptors)	Impaired threat discrimination; lowered threshold for reactive aggression; impulsivity (Zhang-James et al., 2019; Lewis and Picciotto, 2020; Pidoplichko et al., 2013; Titus et al., 2019).	Disrupted theta-band oscillations during affective processing tasks (EEG/MEG)	*Neuromodulation – device-based:* taVNS – partial restoration of NBM-dependent theta rhythmogenesis (Broncel et al., 2018; Song et al., 2025); iTBS – exogenous theta restoration (Huang et al., 2005).
Dopaminergic system (VTA/SNc → amygdala, striatum, PFC; D1/D2 receptors)	Impulsive aggression (hypofunction); instrumental/appetitive aggression and sensation-seeking (D2 dysfunction); impaired cost–benefit computation (Schlüter et al., 2013; Plavén-Sigray et al., 2014; Lukkarinen et al., 2024; Sarkar and Wrangham, 2023).	Reduced subcortical dopamine synthesis/binding ([18F]-FDOPA PET); Impaired reward-guided decision-making tasks	*Pharmacological:* Amantadine (Alderman, 2003; Kangas and McDonald, 2011; Jeffay et al., 2023); PEA-OXA – preclinical, ↑ mPFC dopamine release (Boccella et al., 2020).
Serotonergic system (DRN → OFC/ACC/amygdala/temporal cortex; 5-HT_1A_/_1F_/_2C_, SERT)	Impulsive/reactive aggression; low CSF 5-HIAA; affective dysregulation (Cupaioli et al., 2021; Virkkunen et al., 1995; Jorge and Robinson, 2003; Coccaro, 1989; da Cunha-Bang and Knudsen, 2021)	Decreased CSF 5-HIAA levels; Elevated SERT/5-HT receptor density (PET)	*Pharmacological:* SSRIs (Jorge and Robinson, 2003; Luauté et al., 2016); lithium (Sheard et al., 1976; Leeds et al., 2014; Aswani et al., 2022); risperidone and other atypical antipsychotics (Deb et al., 2020); carbamazepine/valproate (Hammond et al., 2021).
Noradrenergic system (LC → BLA/CeA; α2/β-adrenoceptors)	Reactive aggression with autonomic hyperarousal (LC–amygdala overactivation); chronic hypervigilance/anxiety; blunted in instrumental aggression (Buffalari and Grace, 2007; McCall et al., 2017; Llorca-Torralba et al., 2019; Haden and Scarpa, 2007; Giustino and Maren, 2018).	Elevated autonomic reactivity (HRV, galvanic skin response); Hyper-responsive amygdala BOLD signal (fMRI)	*Pharmacological:* Beta-adrenergic blockers (Hicks et al., 2019; Pisa et al., 2015; Mehta et al., 2018); PEA-OXA – α2-adrenergic antagonist, preclinical (Boccella et al., 2020); *Neuromodulation – device-based:* taVNS (Howland, 2014; Huang et al., 2023; Yakunina et al., 2017; Broncel et al., 2018; Kraus et al., 2007; Song et al., 2025).
MeA–BNST/MeA–Hypothalamus circuits (posterodorsal MeA→BNST; posteroventral MeA→hypothalamus outputs)	Attack initiation and social-approach gating (MeA–BNST); intensity, persistence, and maintenance of aggressive output (MeA–Hypothalamus) (Miller et al., 2019; Nordman et al., 2020).	Decreased oxytocin (OTR)/vasopressin ( $V_{1a}$ ) receptor binding density in extended amygdala hubs. Elevated baseline salivary cortisol-to-testosterone ratio; neuroinflammatory markers (e.g., microglial activation *via* $\left[11C\right]\text{PBR}28$ PET).	*Neuromodulation – device-based:* DBS of the posterior hypothalamus (Contreras Lopez et al., 2021; Franzini et al., 2005); DBS of the BNST (Lebow and Chen, 2016; Lischinsky and Lin, 2020).

Unless otherwise specified, behavioral associations refer to the reactive/impulsive aggression phenotype that is the primary focus of the source review (see Box 2); contrasts with proactive/instrumental aggression are noted where relevant. Within the Candidate Therapeutic Intervention(s) column, “device-based” neuromodulation denotes exogenous stimulation (magnetic, electrical, ultrasonic, or vagal), whereas “behavioral/functional” neuromodulation denotes activity-dependent plasticity induced through structured cognitive-behavioral training. Abbreviations are defined in the Glossary section.

Major challenges for clinical translation include: the heterogeneity of lesions in TBI populations, which requires individualized treatment planning based on neuroimaging; the poorly defined optimal timing of intervention along the post-traumatic trajectory; and the largely uncharacterized interactions between neuromodulation and concomitant pharmacotherapy that target the same neurotransmitter systems that determine thresholds for stimulation-induced plasticity. Identification of reliable quantitative EEG biomarkers of theta coherence and fronto-amygdala coupling for prospective patient stratification and real-time monitoring of therapeutic efficacy constitutes the primary methodological priority for the field (Gkintoni et al., 2025; Huidobro et al., 2025). The forensic and ethical dimensions of neuromodulation in judicially mandated patients, especially considering that the target circuits directly mediate voluntary control, require independent oversight systems of the highest level.

## 7 Conclusion

In conclusion, post-traumatic aggression and antisocial behavior represent a multiscale neurobiological failure where large-scale network desynchronization operates in synergy with localized cellular and neuromodulatory dysregulations within specific amygdalar subnuclei. By charting the precise neurobiological pathways behind these phenotypes, this review fosters a dialogue between traumatic brain injury models, DSM-5-TR taxonomy, and the profound legal dilemmas surrounding volitional control and free will in organically compromised patients. Ultimately, shifting the therapeutic paradigm away from non-specific pharmacological sedation toward personalized, state-dependent neuromodulation and quantitative biomarker strategies offers a precise translational roadmap to restore circuit synchrony, alleviate behavioral distress, and substantively enhance the long-term quality of life for this vulnerable population.

## Glossary

ACC: Anterior Cingulate Cortex
ACh: AcetylCholine
ADPs: current-evoked AfterDePolarizations
aINS: anterior INSula
ASPD: AntiSocial Personality Disorder
BA: Basal Amygdala
BLA: BasoLateral Amygdala
BNST: Bed Nucleus of the Stria Terminalis
CBT: Cognitive Behavioral Therapy
CCK: CholeCystoKinin
CeA: Central Amygdala
CeC: Capsular part of Central amygdala
CeL: Lateral part of Central amygdala
CeM: Medial part of Central amygdala
CEN: Central Executive Network
CRF: Corticotropin-Releasing Factor
CSF: CerebroSpinal fluid
DAI: Diffuse Axonal Injury
DBS: Deep Brain Stimulation
dlPFC: dorsolateral PreFrontal Cortex
DMN: Default Mode Network
DRN: Dorsal Raphe Nucleus
DSM-5-TR: Diagnostic and Statistical Manual of Mental Disorders, Fifth Edition, Text Revision
DTI: Diffusion Tensor Imaging
E/I: Excitation-Inhibition
EPSPs: Excitatory Postsynaptic Potentials
HT: HypoThalamus
5-HT: 5-hydroxytryptamine (serotonin)
IED: Intermittent Explosive Disorder
INS: INSula
IPSPs: Inhibitory PostSynaptic Potentials
ITCs: InTercalated cell Clusters
LA: Lateral Amygdala
LC: Locus Coeruleus
LN: Limbic Network
LNM: Lesion Network Mapping
LTP: Long-Term Potentiation
MACM: Meta-Analytic Connectivity Modeling
MeA: Medial Amygdala
mPFC: medial PreFrontal Cortex
MTBI: mild Traumatic Brain Injury
NBM: Nucleus Basalis of Meynert
NE: NorEpinephrine
OFC: OrbitoFrontal Cortex
PAG: PeriAqueductal Grey
PEA-OXA: 2-PEntAdecyl-2-OXAzoline
PCC: Posterior Cingulate Cortex
PCDAMC: Personality Change Due to Another Medical Condition
PET: Positron Emission Tomography
PV: ParValbumin
rTMS: repetitive Transcranial Magnetic Stimulation
SN: Salience Network
SOM: SOMatostatin
SSRIs: Serotonin Reuptake Inhibitors
taVNS: transcutaneous auricular Vagus Nerve Stimulation
tDCS: transcranial Direct Current Stimulation
TBI: Traumatic Brain Injury
TBS: Theta-Burst Stimulation
tFUS: transcranial Focused UltraSound
vHPC: ventral HiPpoCampus
vmPFC: ventromedial PreFrontal Cortex
VNS: Vagus Nerve Stimulation
VTA: Ventral Tegmental Area
